# Comparing and Validating Methods of Reading Instruction Using Behavioural and Neural Findings in an Artificial Orthography

**DOI:** 10.1037/xge0000301

**Published:** 2017-04-20

**Authors:** J. S. H. Taylor, Matthew H. Davis, Kathleen Rastle

**Affiliations:** 1 Department of Psychology, Royal Holloway, University of London; 2 Medical Research Council Cognition and Brain Sciences Unit, Cambridge, United Kingdom; 3 Department of Psychology, Royal Holloway, University of London

**Keywords:** reading acquisition, reading comprehension, phonics, fMRI, artificial language learning

## Abstract

There is strong scientific consensus that emphasizing print-to-sound relationships is critical when learning to read alphabetic languages. Nevertheless, reading instruction varies across English-speaking countries, from intensive phonic training to multicuing environments that teach sound- and meaning-based strategies. We sought to understand the behavioral and neural consequences of these differences in relative emphasis. We taught 24 English-speaking adults to read 2 sets of 24 novel words (e.g., /buv/, /sig/), written in 2 different unfamiliar orthographies. Following pretraining on oral vocabulary, participants learned to read the novel words over 8 days. Training in 1 language was biased toward print-to-sound mappings while training in the other language was biased toward print-to-meaning mappings. Results showed striking benefits of print–sound training on reading aloud, generalization, and comprehension of single words. Univariate analyses of fMRI data collected at the end of training showed that print–meaning relative to print–sound relative training increased neural effort in dorsal pathway regions involved in reading aloud. Conversely, activity in ventral pathway brain regions involved in reading comprehension was no different following print–meaning versus print–sound training. Multivariate analyses validated our artificial language approach, showing high similarity between the spatial distribution of fMRI activity during artificial and English word reading. Our results suggest that early literacy education should focus on the systematicities present in print-to-sound relationships in alphabetic languages, rather than teaching meaning-based strategies, in order to enhance both reading aloud and comprehension of written words.

There has been persistent and intense debate concerning the manner in which children should be taught to read. There is now a strong scientific consensus regarding the critical importance of instruction that emphasizes the relationship between spelling and sound in reading acquisition (at least in alphabetic writing systems; e.g., National Reading Panel, 2000; Rayner et al., 2001; Rose, 2006). However, the extent to which this scientific consensus around phonic knowledge has permeated policy and practice in the area of reading instruction varies considerably across English-speaking countries. Specifically, while some countries mandate the use of systematic phonics in classrooms, others place greater emphasis on meaning-based knowledge, or use less-structured approaches that include some combination of both. Such differences in emphasis have consequences since, when there is limited instructional time, emphasizing one set of skills necessarily reduces emphasis on another set of skills.

In the present research, we seek to determine the behavioral and neural consequences of forms of reading instruction that emphasize the relationship between print and sound versus the relationship between print and meaning. To do so, we use a laboratory analogue of reading acquisition, in which adults are trained over an extended period on precisely defined novel materials, using precisely defined teaching and testing regimes. By combining this highly controlled method with neuroimaging, we are able to evaluate not only *whether* a manipulation improves reading performance behaviorally (using a range of reading aloud and comprehension tasks), but also *how* it improves performance, in terms of the neural systems modified by a particular experimental manipulation. In this way, our work also demonstrates how neuroscientific evidence can form part of the evidence base for developing educational methods.

## Reading Instruction in Alphabetic Writing Systems

In England, the provision of systematic phonics instruction is a legal requirement in state-funded primary schools. To ensure compliance in all schools, children are required to participate in a national “phonics screen” in their second year of reading instruction (when they are five or six years-old), which measures word and nonword reading aloud. Since its implementation in 2012, the results of this assessment have shown dramatic year-on-year gains in the percentage of children reaching the expected standard—from 58% in 2012 to 81% in 2016. However, despite the apparent success of this policy, there continues to be resistance to it among teachers’ unions and others, who argue in favor of a less-prescriptive approach consisting of a variety of phonic- and meaning-related skills (Association of Teachers & Lecturers, 2016; National Union of Teachers, 2015). One frequent objection is that while phonics may assist reading aloud, it may not promote (and may even erode) reading comprehension (Davis, 2013).

The provision of systematic phonics instruction is also part of the Common Core State Standards Initiative in the U.S. (http://www.corestandards.org/). However, at present, not all U.S. states have adopted the Common Core standards. Further, unlike in England, there is no national assessment of phonic knowledge in young children that could reveal the success of the standards, or individual schools’ compliance with them. Similarly, even among states that have adopted the Common Core standards, there are reports that particular school boards promote “balanced literacy” approaches that include a variety of meaning-related as well as phonic-related skills (Hernandez, 2014; Moats, 2000).

Finally, although reading using phonic knowledge is included in the Australian curriculum, it is suggested as only one strategy, alongside a multicuing approach based on contextual, semantic, and grammatical information (Snow, 2015), including guessing the pronunciation of a word based on a picture or the word’s first letter (Neilson, 2016). The use of systematic phonics instruction is even less widespread in New Zealand classrooms, where text-based information (e.g., predictions based on pictures, preceding context, and prior knowledge) is regarded as more important than word-level phonic information for reading acquisition (Tunmer, Chapman, Greasy, Prochnow, & Arrow, 2013).

This brief review suggests that there is considerable variability in how reading is taught in English-speaking countries. Some prioritize print-to-sound knowledge, others prioritize print-to-meaning knowledge, and still others teach a variety of sound-based and meaning-based skills in the initial periods of reading instruction. Though there is strong evidence for the importance of learning to appreciate print-to-sound relationships in reading acquisition (e.g., National Reading Panel, 2000; Rayner et al., 2001; Rose, 2006), there is limited data on the behavioral and neural consequences of the relative difference in emphasis that characterizes reading instruction in the classroom. In the next section, we consider what might be the cognitive foundations of this differential emphasis.

## Print-to-Sound and Print-to-Meaning Pathways for Reading

The Simple View of Reading (Gough & Tunmer, 1986) has had substantial impact on policy and practice in literacy education (e.g., Rose, 2006). The Simple View proposes that reading comprehension arises from the combination of print-to-sound *decoding* plus oral language skill (i.e., sound-to-meaning mappings), and hence emphasizes the importance of phonic knowledge in reading instruction. However, the Simple View is not a processing model, so is silent as to the actual mechanisms that underpin the discovery of meaning from the printed word. In order to capture these mechanisms, we must look to computational models of reading such as the Dual Route Cascaded model (DRC model, Coltheart, Rastle, Perry, Langdon, & Ziegler, 2001) and the Triangle model (Harm & Seidenberg, 2004; Plaut, McClelland, Seidenberg, & Patterson, 1996).

Like the Simple View of Reading, both the DRC and the Triangle model propose that reading comprehension can be achieved via an indirect pathway that maps from print-to-sound, and then from sound-to-meaning using preexisting oral language. The importance of the print-to-sound-to-meaning pathway is demonstrated by decades of research showing that phonological information is computed rapidly and as a matter of routine in the recognition and comprehension of printed words (Frost, 1998; Rastle & Brysbaert, 2006). In alphabetic and syllabic writing systems, print-to-sound mappings are largely systematic, allowing words to be broken down into symbols that correspond to sounds. The print-to-sound-to-meaning pathway has therefore sometimes been termed the sub-word pathway because of the componential nature of print-to-sound mappings (Wilson et al., 2009; Woollams, Lambon Ralph, Plaut, & Patterson, 2007). However, because the models vary in how they accomplish this mapping, we refer to the indirect pathway from print-to-sound-to-meaning simply as the *phonologically mediated pathway*.

Unlike the Simple View of Reading, both the DRC and Triangle model also propose a direct pathway from print-to-meaning. In alphabetic and syllabic writing systems, in contrast to the relationship between print and sound, the relationship between print and meaning is largely arbitrary and holistic. Similarly spelled words do not have similar meanings (at least when they are morphologically simple), and thus there is no sense in which a word can be broken down into component parts in order to access its meaning. This pathway is therefore sometimes termed the whole-word pathway (Wilson et al., 2009; Woollams et al., 2007). However, the distributed nature of these mappings means that the Triangle model can also capture subword regularities between print and meaning where these exist, for example, in polymorphemic words (Plaut & Gonnerman, 2000). Thus, throughout this article we will refer to the mapping from print-to-meaning as the *direct pathway*, because both models propose that written words can be comprehended without phonological mediation.

Plaut et al. (1996) argued that the “division of labor” between the phonologically mediated versus the direct pathway between print and meaning depends on the necessity of these processes for producing the appropriate response, given the task being performed and the characteristics of the orthography. This has led to suggestions that the direct pathway may only be necessary in orthographies with some degree of inconsistency between spelling and sound, because otherwise the phonologically mediated pathway can support accurate reading aloud and comprehension (Share, 2008; Ziegler & Goswami, 2005). However, this overlooks the fact that comprehension of written words should be more efficient using the direct than the phonologically mediated pathway, irrespective of spelling–sound consistency (Seidenberg, 2011). In the current research we therefore sought to determine how reading instruction that emphasizes print-to-sound versus print-to-meaning mappings impacts on the development of these pathways, and thus on reading aloud and comprehension of written words, in a regular orthography.

## Neuroimaging and Neuropsychological Evidence for Dual Reading Pathways

Neuroimaging and neuropsychological evidence offer strong support for the notion of dual pathways to meaning proposed in cognitive models. This evidence has yielded a model in which phonologically mediated reading is underpinned by a *dorsal* pathway including left posterior occipitotemporal cortex, inferior parietal sulcus, and dorsal portions of the inferior frontal gyrus (opercularis, triangularis). Data from fMRI experiments in alphabetic languages reveal that these regions consistently show greater activation for nonwords than words (Taylor, Rastle, & Davis, 2013). Left inferior parietal cortex has also been found to be more active when reading alphabetic relative to logographic writing systems (Bolger, Perfetti, & Schneider, 2005). Furthermore, patients with damage to left posterior occipitotemporal cortex show slow and effortful reading in alphabetic scripts (Roberts et al., 2013), and those with damage to left inferior parietal cortex and dorsal inferior frontal gyrus show poor nonword relative to word reading (Rapcsak et al., 2009; Woollams & Patterson, 2012).

Conversely, neural evidence suggests that *ventral* pathway brain regions underpin direct print-to-meaning processes. Vinckier et al. (2007) showed that from left posterior to anterior occipitotemporal cortex there is an increasingly graded response to the word-likeness of written stimuli, with the mid-fusiform/inferior temporal gyrus responding more strongly to words and pseudowords than to stimuli containing frequent bigrams, followed by consonant strings, then false fonts. This processing hierarchy is supported by analyses of anatomical connectivity (Bouhali et al., 2014), with posterior occipitotemporal cortex connecting to speech processing regions such as left inferior frontal gyrus and posterior middle and superior temporal gyri, whereas anterior fusiform shows connectivity with more anterior temporal regions that are important for semantic processing. Supporting the idea that direct print-to-meaning processes are underpinned by anterior fusiform, meta-analytic fMRI data reveal greater activation for words than nonwords in this region, in addition to more lateral temporal lobe regions such as left middle temporal and angular gyri^1^ (Taylor et al., 2013). Similarly, patients with left anterior fusiform lesions display poorer performance in reading and spelling words than nonwords (Purcell, Shea, & Rapp, 2014; Tsapkini & Rapp, 2010). Further evidence for the correspondence between the direct pathway and ventral brain regions comes from semantic dementia patients, who have atrophy in anterior temporal lobes, including anterior fusiform gyrus (Mion et al., 2010), and often show particular problems reading aloud words with atypical spelling-to-sound mappings (Woollams et al., 2007). These irregular or inconsistent words depend more heavily on the direct pathway than words with typical or consistent print-to-sound relationships, which primarily rely on the phonologically mediated pathway.

## The Development of Neural Reading Pathways

Like adults, children show neural activity in both dorsal and ventral pathways for a simple contrast of reading words and/or nonwords relative to rest (Martin, Schurz, Kronbichler, & Richlan, 2015). Supporting the involvement of dorsal stream regions in children’s phonologically mediated reading skills, activity in left dorsal inferior frontal gyrus and inferior parietal cortex during a rhyme judgement task was positively correlated with change in nonword reading skill, between the ages of 9 and 15 (McNorgan, Alvarez, Bhullar, Gayda, & Booth, 2011). Providing further longitudinal evidence, Preston et al. (2016) showed that in left fusiform gyrus, inferior parietal cortex, and dorsal inferior frontal gyrus, the degree of convergence between neural activity during print and speech tasks at age 8 predicted reading skill at age 10.

Computational models suggest that the involvement of the direct pathway increases with reading skill (Harm & Seidenberg, 2004; Plaut et al., 1996). In line with this, several authors have proposed that as children become better readers, reliance shifts from the dorsal to the ventral pathway (Pugh et al., 2000; Rueckl & Seidenberg, 2009; Sandak et al., 2012). This conceptualization is supported by longitudinal data showing that areas of the ventral pathway increase in sensitivity to written words between the ages of 9 and 15, and that this increasing sensitivity is associated with speeded word reading ability but not with nonword reading or phonological processing skill (Ben-Shachar, Dougherty, Deutsch, & Wandell, 2011).

Overall, although the data from children are somewhat limited, they support the proposed distinction between the phonologically mediated dorsal pathway and the direct print-to-meaning ventral pathway. However, we are unaware of any evidence linking instructional methods to changes in these neural systems.

## Laboratory Approaches to Studying Language Learning

Ultimately, the questions being addressed in this manuscript need to be investigated in child populations. However, to provide an initial investigation into the impact of teaching method on neural mechanisms for reading, we used an artificial language approach with adults. There has been a surge of interest in recent years in using these approaches to model the acquisition of different types of linguistic information (Bowers, Davis, & Hanley, 2005; Clay, Bowers, Davis, & Hanley, 2007; Fitch & Friederici, 2012; Gaskell & Dumay, 2003; Hirshorn & Fiez, 2014; Tamminen, Davis, & Rastle, 2015; Taylor, Plunkett, & Nation, 2011). In contrast to studying children learning their first language (who vary in their prior experience with both spoken and written forms), artificial language approaches provide total control over participants’ prior knowledge of a new language or writing system. They also make it possible to manipulate what participants are taught and how they are taught in a way that could never be achieved in a naturalistic learning setting with children. Finally, working with adult learners permits collection of more extensive behavioral and brain imaging evidence during different stages of acquisition than would be possible with children. Artificial language learning studies consistently show that participants can learn sets of novel linguistic materials to a high degree of accuracy in a single training session, that this knowledge is sufficient to promote generalization to untrained materials (Tamminen et al., 2015; Taylor et al., 2011; Taylor, Rastle, & Davis, 2014a), and that this knowledge is long lasting (Havas, Waris, Vaquero, Rodríguez-Fornells, & Laine, 2015; Laine, Polonyi, & Abari, 2014; Merkx, Rastle, & Davis, 2011; Tamminen & Gaskell, 2008).

This body of research has yielded interesting insights into the mechanisms that underpin the learning and abstraction of different types of linguistic information. However, significant questions remain over the extent to which artificial language learning reflects natural acquisition processes. Indeed, in the related field of artificial grammar learning, there is a long history of debate over the nature of knowledge acquired (Perruchet & Pacteau, 1990; Reber, 1967; see Pothos, 2007, for a review). For example, if these paradigms reflect strategic, problem solving operations rather than the development of long-term abstract knowledge, then this would undermine their usefulness in understanding the acquisition of linguistic knowledge. Similarly, it could be that adults who have fully developed language and/or reading systems solve these laboratory learning tasks in fundamentally different ways than children acquiring these linguistic skills for the first time. One approach to countering these criticisms has been to demonstrate that similar behavioral effects emerge in artificial language learning studies as in natural languages (e.g., frequency and consistency effects on reading aloud; Taylor et al., 2011), while another has been to show that the constraints that underpin artificial language learning in adults also pertain to children (Henderson, Weighall, Brown, & Gaskell, 2013). However, more direct evidence that the processes recruited in artificial language learning paradigms overlap with those used in natural language would be desirable.

In this article we propose that neuroimaging data may provide this more direct evidence. Specifically, we will use brain responses to gain information about the mechanisms underlying different methods of literacy instruction in alphabetic writing systems. This approach is appropriate and timely because, as outlined earlier, the neural systems that underpin the phonologically mediated and direct pathways to reading in adults are well understood and appear to be similar in children (Martin et al., 2015; Taylor et al., 2013). We propose that we can capitalize on knowledge of these pathways to assess (a) whether training people to read new words printed in artificial scripts engages these neural reading pathways, and (b) whether and how different parts of the reading system are affected by different forms of training. These observations would provide direct evidence not only of the value of artificial learning paradigms for investigating reading, but also of the mechanism by which a particular training intervention operates. We believe that these inferences would be very difficult to draw from a purely behavioral outcome (e.g., accuracy or learning rate in a particular training condition), or through naturalistic study of children learning to read in their first language. This knowledge gained from our study will therefore complement that acquired from studies conducted on children, to inform the development of new interventions that target specific reading pathways.

## Laboratory Approaches to Studying the Neural Basis of Reading Acquisition

In the present study we aimed to uncover the neural consequences of reading instruction that prioritizes print-to-sound versus print-to-meaning mappings. Previous training studies suggest that learning and retrieving componential print-to-sound associations for novel words (written either in familiar or artificial letters) modulates neural activity in dorsal pathway brain regions such as left inferior parietal cortex and inferior frontal gyrus (Mei et al., 2014; Quinn, Taylor, & Davis, 2016; Sandak et al., 2004; Taylor et al., 2014a). However, modulation of ventral pathway activity in artificial language learning studies has been somewhat elusive. Taylor et al. (2014a) found that learning whole object names activated the ventral pathway (left anterior fusiform gyri and ventral inferior frontal gyrus), more than learning letter-to-sound associations (see also, Quinn et al., 2016, for a similar finding when retrieving object names). However, no studies have reported ventral pathway activity for training on whole written words; instead, left angular and middle temporal gyri are more often implicated (Mei et al., 2014; Takashima et al., 2014). The failure to observe ventral pathway activity for trained words may be the result of relatively short and/or superficial training regimes, or because trained words were meaningless. In the current study, we therefore trained novel words extensively, all items had associated meanings, and we examined dorsal and ventral pathway activation during both phonological and meaning based tasks.

## The Present Study

**Figure 1 fig1:**
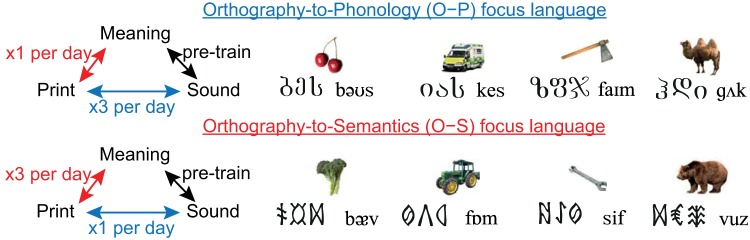
Examples of stimuli used and illustration of how the learning focus manipulation was implemented. Note that this represents the experience of one participant as the assignment of orthography to spoken word set, noun set, and learning focus was counterbalanced across participants, as detailed in Appendix B. See the online article for the color version of this figure.

**Figure 2 fig2:**
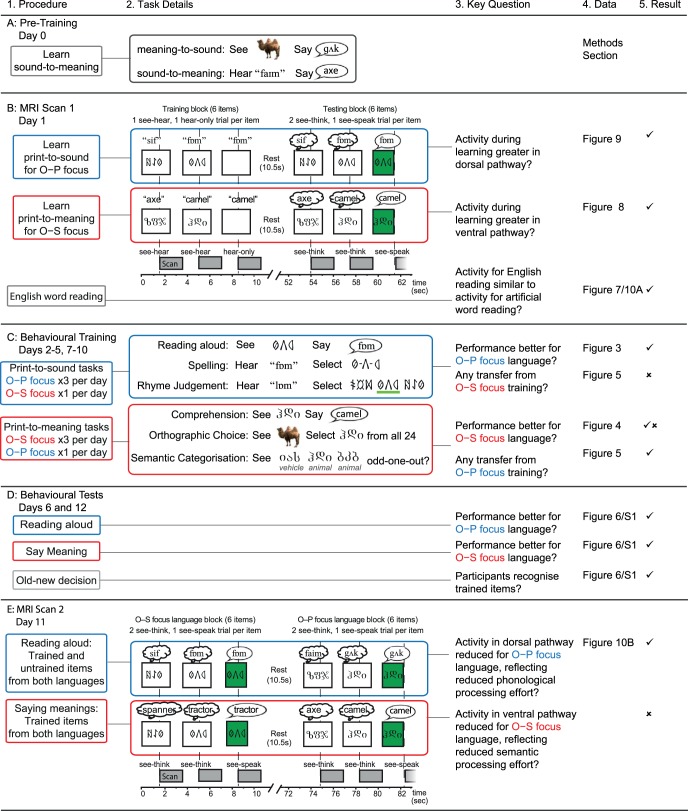
Overview of procedures, task details, key questions, data sets, and results for each part of the experiment. Column 1 gives an overview of each training procedure. Column 2 gives further details of the behavioral and MRI protocols. In rows B and E, which show the trial format and timing during MRI scans, dotted lines indicate correspondence between stimulus presentation and scan onset. Black outlined boxes show what participants were viewing, and what they were hearing, thinking, or saying is shown above each box. Column 3 delineates the key questions addressed by each part of the experiment, column 4 shows where the results can be found, and in column 5 ticks and crosses indicate whether each prediction was confirmed by the data. See the online article for the color version of this figure.

We used an artificial language paradigm underpinned by fMRI measures of brain activity to reveal the behavioral and neural consequences of an emphasis on print-to-sound versus print-to-meaning mappings as adults learned to read new alphabetic orthographies. We used a within-subject design in which 24 adults learned to read two sets of novel words (henceforth referred to as languages) written in two different sets of unfamiliar symbols (orthographies), over a 2-week training period. Figure 1 provides some examples of the stimuli, and Appendix A shows the stimuli learned by one participant. Participants were first preexposed to the sounds (phonology) and meanings (semantics) of the novel words in each language (Figure 2, row A). They then learned both orthography-to-phonology (O–P, print-to-sound) and orthography-to-semantic (O–S, print-to-meaning) mappings for each language over a 2-week training period (Figure 2, row C). Each orthography had a systematic one-to-one correspondence between print and sound, and an arbitrary whole-word correspondence between print and meaning. Our artificial languages therefore had writing systems that were similar to those of natural languages with transparent orthographies, such as Spanish or Italian. However, we manipulated the focus of learning: for one language participants received three times as much training on O–P mappings and for the other they received three times as much training on O–S mappings.[Fig-anchor fig1][Fig-anchor fig2]

All aspects of the stimulus sets were counterbalanced across subjects, including which set of meanings was associated with which set of spoken words, which set of spoken words was written in which orthography, and which training focus was associated with which orthography (full counterbalancing details provided in Appendix B). Thus, any observed influences of training focus on learning could not be attributed to any inadvertent differences between the sets of spoken words, symbol sets, or meanings.

We measured behavioral performance throughout the course of training, when the relative amount of exposure to orthography–phonology and orthography–semantic mappings varied between the two languages. Literacy acquisition in this laboratory model thereby enabled us to assess the behavioral consequences of an emphasis on print-to-sound versus print-to-meaning relationships for reading aloud and comprehension of printed words. We also used brain imaging to assess the neural impact of the different training protocols in three different ways.

1 The existing literature suggests that learning print-to-sound and print-to-meaning mappings should engage the dorsal and ventral pathways of the reading network respectively. To determine whether this was indeed the case, we measured neural activity while participants learned print-to-sound mappings for the O–P focus language and print-to-meaning mappings for the O–S focus language. This was participants’ first exposure to the two artificial orthographies. Figure 2, row B provides further details about MRI Scan 1, which also included an English word and pseudoword reading task.
2 Following 2-weeks of intensive behavioral training, participants underwent a second scanning session (MRI Scan 2), in which they generated pronunciations (reading aloud) and meanings (reading comprehension) of trained items from both languages. Details of these tasks are provided in Figure 2, row E. This enabled us to examine whether the two training regimes (O–P vs. O–S focus) differentially impacted activity in the dorsal and ventral pathways of the reading network during both reading aloud and reading comprehension. We anticipated that training focused on print-to-sound, rather than print-to-meaning, mappings should increase the efficiency of the phonologically mediated pathway. Thus, by the end of training, activity in brain regions along the dorsal pathway (e.g., inferior parietal sulcus, inferior frontal gyrus) should be reduced during reading aloud, reflecting less effortful processing for the print-to-sound than the print-to-meaning focused language. In the context of this artificial language, we can also address whether the converse is true—that is, whether training focused on print-to-meaning mappings increases the efficiency of the direct pathway. If so, then by the end of training, we would expect activity in brain regions along the ventral pathway (e.g., anterior fusiform gyrus) to be reduced during reading comprehension, indicating less effortful processing for the print-to-meaning than the print-to-sound focused language. Such an outcome could indicate that there are positive neural consequences of “balanced literacy” reading instruction programs that emphasize print-to-meaning relationships.
3 In MRI Scan 2, participants also read aloud untrained items from both the artificial languages. We used these data to compare the magnitude and spatial distribution of neural activity when reading aloud trained and untrained items from the artificial languages to that seen for English word and pseudoword reading (English data collected in MRI Scan1). This enabled us to determine the extent to which neural activity when reading the trained orthographies resembled reading in natural language, and thus to assess the ecological validity of using artificial orthographies to test hypotheses about literacy acquisition.

## Method

### Participants

Twenty-four native English speaking adults (21 females) aged 18–30 participated in this study. None of the participants had any current, or history of, learning disabilities, hearing impairments, or uncorrected vision impairments. All participants were right-handed and were students or staff at Royal Holloway, University of London, United Kingdom. Participants were paid for their participation in the study.

### Stimuli

#### Spoken pseudoword forms

Six sets of 24 monosyllabic consonant-vowel-consonant (CVC) pseudowords were constructed from 12 consonant (/b/, /d/, /f/, /g/, /k/, /m/, /n/, /p/, /s/, /t/, /v/, /z/) and eight vowel phonemes; four vowels occurred in three sets of pseudowords (language 1: /ε/, /ʌ/, /aɪ/, /əʊ/) and four occurred in the other three sets (language 2: /æ/, /ɒ/, /i/, /u/). The two languages were constructed using different vowel phonemes to minimize their confusability, because each participant learned one set of items from Language 1, and another set of items from Language 2. Within each set of pseudowords, consonants occurred twice in onset position, and twice in coda position, whereas vowels occurred six times each. Pseudowords were recorded by a female native English speaker and digitized at a sampling rate of 44.1 kHz. For each participant, one set of pseudowords from each language constituted the trained items, one set from each language was used as untrained items to test generalization at the end of training, and one set from each language was used as untrained items for old-new decision to test whole-item recognition at the end of training. The assignment of pseudoword sets to conditions (trained, generalization, old-new decision) was counterbalanced across participants, as detailed in Appendix B.

#### Artificial orthographies

Two sets of 20 unfamiliar alphabetic symbols were selected from two different archaic orthographies (Hungarian Runes, Georgian Mkhedruli). Each phoneme from the two languages was associated with one symbol from each orthography, for example, the phoneme /b/ was associated with one Hungarian symbol and one Georgian symbol. Thus, there was an entirely regular, or one-to-one correspondence between symbols and sounds in each orthography. Each participant learned to read a set of trained items from one language written in Hungarian Runes and a set of trained items from the other language written in Georgian Mkhedruli. The assignment of language to orthography was counterbalanced across participants, as detailed in Appendix B. Some examples of items written in the artificial orthographies are shown in Figure 1, and the full set of items learned by one participant is shown in Appendix A.

#### Word meanings

Two sets of 24 familiar objects, comprising six fruits and vegetables, six vehicles, six animals, and six tools, were assigned to the two sets of trained items. Each item had an associated picture and a single word name. The majority of these items were used in experiments by Tyler et al. (2003). The two sets were matched on familiarity using the MRC Psycholinguistic database (Coltheart, 1981). A photo of each item was selected from the Hemera Photo Objects 50,000 Premium Image Collection, or where this was not possible (*n* = 9) from the Internet. Trained items were assigned meanings arbitrarily, such that there was no systematic relationship between the sound or spelling of each item and its meaning. The assignment of noun set to language, orthography, and training focus, was counterbalanced across participants, as detailed in Appendix B. Example stimuli are shown in Figure 1.

#### English words and pseudowords

At the end of the first scanning session, participants read aloud 60 regular words and 60 irregular words, half of which were high and half of which were low in imageability, and 60 pseudowords. We used these data to establish the extent to which neural activity during reading of the artificial languages was similar to that seen during English word and pseudoword reading. Regularity was defined using the grapheme-to-phoneme rules implemented in the DRC model (Coltheart et al., 2001). The four sets of words were pairwise matched on length, phonetic class of onset, log frequency from the Zipf scale (van Heuven, Mandera, Keuleers, & Brysbaert, 2014) and orthographic neighborhood size from the English Lexicon Project (Balota et al., 2007). Words and pseudowords were matched on length, phonetic class of onset, and orthographic neighborhood size (details provided in Appendix C).

### Procedure

#### Phonology-to-semantic pretraining (see Figure 2A)

Before beginning the orthography training, participants learned the association between the spoken forms of the 24 trained pseudowords from each language and their meanings. Items from the two languages were presented in separate alternating training runs, and participants completed three runs for each language. In each run there were eight alternating blocks of training and testing. In each of the four training blocks, participants listened to six pseudowords, each presented with a picture of a familiar noun alongside its written English name. Each training block was followed by two testing blocks: one in which participants saw pictures of the six nouns they had just learned and overtly produced the associated pseudowords (semantic-to-phonology), and another in which they heard the six pseudowords they had learned and had to say the English translation (phonology-to-semantic). For trained items that contained the vowels /ε/, /ʌ/, /aɪ/, /əʊ/ (Language 1), the mean proportion of items correctly recalled by the end of the third run was .69 (within-subject standard error (*SE*) = .03) for semantic-to-phonology mappings, and .84 (*SE* = .02) for phonology-to semantic mappings. For the trained items that contained the vowels /æ/, /ɒ/, /i/, /u/ (Language 2), semantic-to-phonology mapping accuracy by Run 3 was .80 (*SE* = .02) and phonology-to-semantic mapping accuracy was .88 (*SE* = .03). Note that, due to counterbalancing procedures detailed in Appendix B, these slight differences in performance for the two languages could not have impacted on subsequent performance for O–P versus O–S focused training.

Due to adjustments made to scheduling, phonology-to-semantic pretraining took place the day before the first scan started for 6 participants, but one week before the first scan for 18 participants. These latter participants also completed an additional run of phonology-to-semantic training the day before the first scan. Performance remained stable between the third run of the initial pretraining session and this additional run.^2^ Overall, pretraining provided participants with relatively good knowledge of the relationship between the phonological and semantic forms of the two languages.

#### First MRI scanning session (Figure 2B)

Within the first scanning session, participants learned print-to-sound mappings for the O–P focus language (reading aloud) and print-to-meaning mappings for the O–S focus language (saying the meanings). The assignment of orthography to print-to-sound or print-to-meaning learning was counterbalanced across participants. In a final scanning run they then completed an English word and pseudoword reading aloud task. Further details of the functional imaging acquisition procedures are provided later in the Methods.

##### Print-to-sound and print-to-meaning training

Participants completed two learning runs for the O–P focus language, which involved learning print-to-sound associations, and two for the O–S focus language, which involved learning print-to-meaning associations. All 24 trained items from the language were presented in a randomized order in each run. Run types alternated and half the participants started with O–P learning and half with O–S learning.

Each run was broken down into four training blocks in which participants learned six items; each training block was followed by a test block in which participants retrieved pronunciations or meanings for the six items. Training blocks comprised 12 trials presented in a randomized order. Six had concurrent visual and spoken form presentation (see-hear) and six had isolated spoken form presentation (hear-only). Contrasts between these two trial types enabled us to examine how activity differed when a trial afforded a learning opportunity (see-hear) relative to when it did not (hear-only). For O–P learning runs, spoken forms constituted the pronunciation of the written word in the new language, whereas for O–S learning runs, spoken forms constituted the English meaning of the written word. Each training trial was 3,500 ms in duration, with visual items presented for the first 2,500 ms and spoken forms commencing at the onset of the trial. Scan volume acquisition (2,000 ms) commenced 1,500 ms after the trial onset.

In testing blocks, participants retrieved the pronunciations or meanings learned in the preceding training block. Testing blocks comprised 12 see-think trials, presented in a randomized order, in which participants were presented with an item’s visual form on a white background and covertly retrieved its pronunciation or meaning. Half of the see-think trials were immediately followed by a see-speak trial, in which the same item was presented on a green background and participants overtly articulated its pronunciation or meaning having retrieved it in the preceding see-think trial. This split trial format allowed participants enough time to both retrieve the pronunciation (see-think) and articulate it (see-speak). Each testing trial was 3,500 ms in duration, with visual forms presented at the beginning of the trial and scanning acquisition (2,000 ms) commencing after 1,500 ms. Visual forms were presented for 2,500 ms on see-think trials and 1,500 ms on see-speak trials to encourage participants to generate spoken forms before the onset of scan volume acquisition. Stimuli were presented over high quality etymotic headphones, and responses were recorded using a dual-channel MRI microphone (FOMRI II, Optoacoustics) and scored offline for accuracy. Response times (RTs) were labeled manually through inspection of the speech waveform using CheckFiles (a variant of CheckVocal, Protopapas, 2007). The same recording and scoring procedures were used for all MRI tasks.

##### English word and pseudoword reading

The 180 words and pseudowords were presented in a randomized order and participants were instructed to read each item aloud as quickly and accurately as possible. Items were presented in the center of a white background, in black, 32-point Arial font for 1,500 ms, and each was followed by a 2,000 ms blank screen interstimulus interval. Scan volume acquisition (2,000 ms) commenced at the onset of the blank screen. This scanning run therefore used the same timing as for see-speak trials in test blocks from the O–P and O–S learning runs. Eighteen items were presented per block and each block was followed by a 10.5-s blank screen rest period. Responses were recorded using a dual-channel MRI microphone. Contrasts between words and pseudowords were used to inform interpretations of activity during reading aloud and saying the meanings of items at the end of training.

#### Behavioral training (Figure 2C)

Participants learned about the two novel languages for an average of 1.5 hr per day (tasks were self-paced), for eight consecutive days, with breaks for weekends. To maximize participant engagement and minimize boredom, on each day they engaged in six different tasks for each language, three that involved mapping between orthography and phonology (O–P tasks) and three that involved mapping between orthography and semantics (O–S tasks). The nature of the tasks that involved O–P and O–S learning was matched as closely as possible. We manipulated the focus of learning, such that it prioritized orthography-to-phonology mappings for one language (the one for which they learned print-to-sound mappings in the scanner) and orthography-to-semantic mappings for the other language (the one for which they learned print-to-meaning mappings in the scanner). This manipulation was instantiated as follows: For the O–P focus language, participants completed O–P tasks three consecutive times per day and O–S tasks only once per day. Conversely, for the O–S focus language, participants completed O–S tasks three consecutive times per day and O–P tasks only once per day. Across the days, we varied the order in which the tasks were completed and whether each task was first completed for the O–P or the O–S language. For tasks requiring spoken responses, these were recorded and manually coded offline for accuracy and RT. Accuracy and RTs were recorded by E-Prime for tasks that required keyboard or mouse responses.

##### O–P tasks

The following tasks emphasized learning of mappings between orthography and phonology.

###### Reading aloud

The orthographic forms of each of the 24 trained items were presented in a randomized order. Participants read them aloud, that is, said their pronunciation in the new language, and pressed spacebar to hear the correct answer. This task therefore emphasized mapping from orthography to phonology.

###### Spelling

Participants heard the phonological form of a trained item, while viewing all 16 symbols from the corresponding orthography, presented in a four-by-four array. They then clicked (using the mouse) on the three symbols that spelled that item in the correct order. After each trial, feedback was given indicating which symbols were correct and which were incorrect, and the correct spelling was also displayed alongside the participant’s spelling. This task therefore emphasized mapping from phonology to orthography.

###### Rhyme judgment

On each trial, the orthographic forms of three trained items were presented on the left, center, and right of the screen. Participants heard a pseudoword that rhymed with one of these items and were instructed to press 1 (left), 2 (center), or 3 (right), to indicate which item it rhymed with. They then received feedback as to which was the correct answer and also heard how that item was pronounced. This task required participants to understand the mappings between orthography and phonology but was somewhat easier than the other two O–P tasks due to its forced choice nature. Each trained item was presented once as a target, and the two trained item distractors were pseudorandomly selected such that at least one shared either the final consonant or the vowel phoneme, but neither shared both the vowel and final consonant. In order to ensure that responses did not become overlearned, five rhyming pseudowords were generated for each trained item. Most were generated using Wuggy (Keuleers & Brysbaert, 2010).

##### O–S tasks

The following tasks emphasized learning of mappings between orthography and semantics.

###### Saying the meaning

The orthographic forms of each of the 24 trained items were presented in a randomized order. Participants said their English meaning aloud and then pressed spacebar to hear the correct answer. This task therefore emphasized mapping from orthography to semantics.

###### Orthographic search

Participants saw the orthographic forms of all 24 trained items in a 6 × 4 array and, above this, a picture of one of the trained meanings. Participants used the mouse to click on the orthographic form that matched the meaning and were then given feedback as to which item they should have selected. This task therefore emphasized mapping from semantics to orthography.

###### Semantic categorization

Participants saw the orthographic forms of three trained items and had to press 1 (left), 2 (center), or 3 (right), to indicate which was from a different semantic category from the other two. Participants were told that there were four categories; animals, vegetables/fruit, vehicles, and tools. Each trained item target was presented once, but the distractor category and items from that category were randomly selected on each trial. Feedback was given to indicate the correct answer, alongside a picture of the meaning of that item. This forced choice task was chosen to be of the same nature as the rhyme judgment task, but emphasized the mapping between orthography and semantics rather than orthography and phonology.

#### Second MRI scanning session (Figure 2E)

Neural activity was measured while participants read aloud the 24 trained and 24 untrained items from each language, and said the meanings of the 24 trained items from each language. The reading aloud task was split across two runs as it comprised twice as many items as the saying the meaning task. Half the participants first completed two *reading aloud runs* and then one *saying the meaning run*, whereas half the participants had the reverse task order. Trained and untrained items were presented in a randomized order across the two *reading aloud runs*, and participants were informed that they would be asked to say the pronunciations of items they had learned as well as new items written in the same symbols. Similarly, trained items were presented in a randomized order in the *saying the meaning run*, and participants were instructed to say the English meaning of each item.

Forty-eight items (24 from each language) were presented in each run, and there were two see-think and one see-speak trials for each item. In see-think trials, participants were presented with an item’s artificial orthographic form and covertly retrieved its pronunciation or meaning. Half the see-think trials were immediately followed by a see-speak trial, in which the same item was presented on a green background and participants overtly articulated its pronunciation or meaning, having retrieved it in the preceding see-think trial. Runs were split into blocks of 18 trials (12 see-think and six see-speak), with alternating blocks for the two different languages, to avoid undue confusion between them.

All trials were 3,500 ms in duration, with visual items presented for the first 2,500 ms followed by a blank screen on see-think trials, and for the first 1,500 ms followed by a blank screen on see-speak trials. Scan volume acquisition (2,000 ms) commenced 1,500 ms after the onset of the visual form. A 10.5-s rest period during which a blank screen was presented followed each block. Further details of the functional imaging acquisition procedures are provided later in the Method section.

#### Behavioral testing (Figure 2D)

After four days of training (Day 6), and following the second MRI Scan (Day 12), participants completed several tests to assess learning. These included reading aloud and saying the meanings of trained items, which took exactly the same form as during training, except that only one run of each task was completed, and no feedback was given. Participants were additionally asked to read aloud untrained items, which proceeded in the same way as reading aloud trained items, but participants were informed that the items they were going to be reading would be unfamiliar words written in the same symbols as the words they had been learning. This tested their ability to generalize their knowledge about the symbol-to-sound mappings in each language. They also completed an old-new decision task for each language in which they saw the orthographic forms of 24 trained and 24 untrained items, in a randomized order, and were asked to press Z if they had learned the item, and M if they had not learned the item. This tested whether they recognized the whole-word forms of the trained items. For all of the test tasks, the two languages were presented in separate runs.

### Functional Imaging Acquisition

Functional MRI data were acquired on a 3T Siemens Trio scanner (Siemens Medical Systems, Erlangen, Germany) with a 32-channel head coil. Blood oxygenation level-dependent functional MRI images were acquired with fat saturation, 3 mm isotropic voxels and an interslice gap of .75 mm, flip angle of 78 degrees, echo time [TE] = 30 ms, and a 64 × 64 data matrix. For all tasks, in both scanning sessions, we used a sparse imaging design with a repetition time (TR) of 3,500 ms but an acquisition time (TA) of 2,000 ms, which provided a 1,500-ms period in which to present spoken words and record spoken responses in the absence of echoplanar scanner (Edmister, Talavage, Ledden, & Weisskoff, 1999). The acquisition was transverse oblique, angled to avoid the eyes and to achieve whole-brain coverage including the cerebellum. In a few cases the very top of the parietal lobe was not covered. To assist in anatomical normalization we also acquired a T_1_-weighted structural volume using a magnetization prepared rapid acquisition gradient echo protocol (TR = 2,250 ms, TE = 2.99 ms, flip angle = 9 degrees, 1 mm slice thickness, 256 × 240 × 192 matrix, resolution = 1 mm isotropic).

Six dummy scans were added at the start of each run to allow for T1 equilibration, these scans are excluded from statistical analyses. In the first scanning session, 144 images were acquired in each of the 8.4 min O–P and O–S training runs, and 210 images were acquired in the 12.25 min English word and pseudoword reading run. In the second scanning session, 336 images were acquired across the two 9.8 min reading aloud testing runs, and 168 images were acquired in the 9.8 min say the meaning testing run.

Image processing and statistical analyses were performed using SPM8 software (Wellcome Trust Centre for Functional Neuroimaging, London, United Kingdom). Images for each participant were realigned to the first image in the series (Friston et al., 1995) and coregistered to the structural image (Ashburner & Friston, 1997). The transformation required to bring a participant’s structural T1 image into standard Montreal Neurological Institute (MNI) space was calculated using tissue probability maps (Ashburner & Friston, 2005) and these warping parameters were then applied to all functional images for that participant. Normalized functional images were resampled to 2 mm isotropic voxels. The data were spatially smoothed with an 8mm full-width half maximum isotropic Gaussian kernel prior to model estimation.

Data from each participant were entered into general linear models for event-related analysis (Josephs & Henson, 1999). In all models, events were convolved with the SPM8 canonical hemodynamic response function (HRF). Movement parameters estimated at the realignment stage of preprocessing were added as regressors of no interest. Low frequency drifts were removed with a high-pass filter (128 s) and AR1 correction for serial autocorrelation was made. Contrasts of parameter estimates were taken forward to second level group analyses (paired-sample *t* tests, repeated measures analysis of variance [ANOVA]) using participants as a random effect. All comparisons were assessed using a voxelwise uncorrected threshold of *p* < .001. After thresholding, only activations exceeding a cluster extent family wise error (FWE) corrected threshold of *p* < .05, obtained using the nonstationarity toolbox in SPM8 (Hayasaka, Phan, Liberzon, Worsley, & Nichols, 2004) were further considered for interpretation. Figures show results at this cluster extent corrected threshold, displayed on a canonical brain image. Cluster coordinates are reported in the space of the MNI152 average brain template and anatomical labels were generated by MRICron (Rorden, Karnath, & Bonilha, 2007) which uses the automated anatomical labeling (AAL) template (Tzourio-Mazoyer et al., 2002). Further details about each of these univariate analyses are provided in the Results section.

In addition to analyses of the magnitude of activation in different conditions, we also analyzed the spatial distribution of activation for particular contrasts. These analyses allowed us to quantify spatial similarity between activation maps for reading artificial and English items (e.g., comparing the contrast of trained vs. untrained artificial items with English words vs. pseudowords). For these analyses, similarity was quantified using voxelwise correlation of T-statistic values for relevant contrasts in single subjects. These methods are similar to those used in Haxby et al. (2001), and used custom Matlab code that was informed by methods developed by Kriegeskorte, Mur, and Bandettini (2008) and Nili et al. (2014). Correlation coefficients in single participants were computed over voxels selected to fall within gray-matter masks defined by thresholding tissue probability maps derived from the normalization stage of preprocessing. Pearson correlation coefficients derived from statistical maps in single participants were Fisher Z-transformed to conform to normality assumptions and then entered into one-sample *t* tests over participants to compare observed correlations to the null hypothesis.

## Results

### Performance During Training

#### Orthography–phonology and orthography–semantic training

**Figure 3 fig3:**
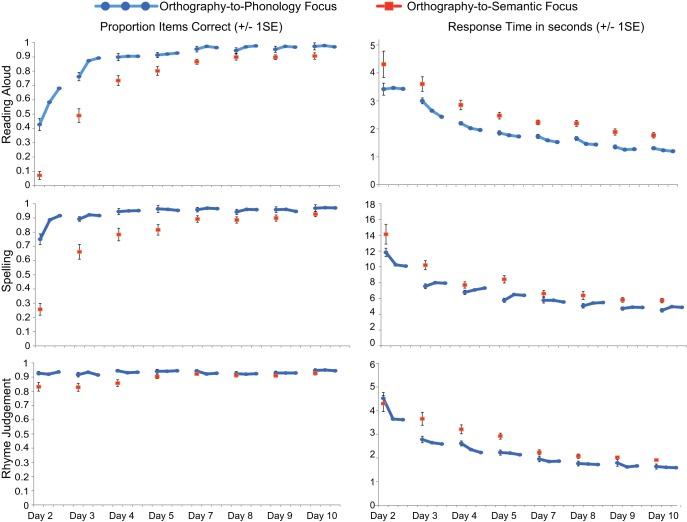
Accuracy and RTs in tasks that involved mapping between orthography and phonology for the O–P and O–S focus languages on each day and each session of training. All error bars in this and subsequent figures use standard error appropriate for within-participant designs (Loftus & Masson, 1994). Error bars are shown only for the first session on each day because only these data points were statistically compared. See the online article for the color version of this figure.

**Figure 4 fig4:**
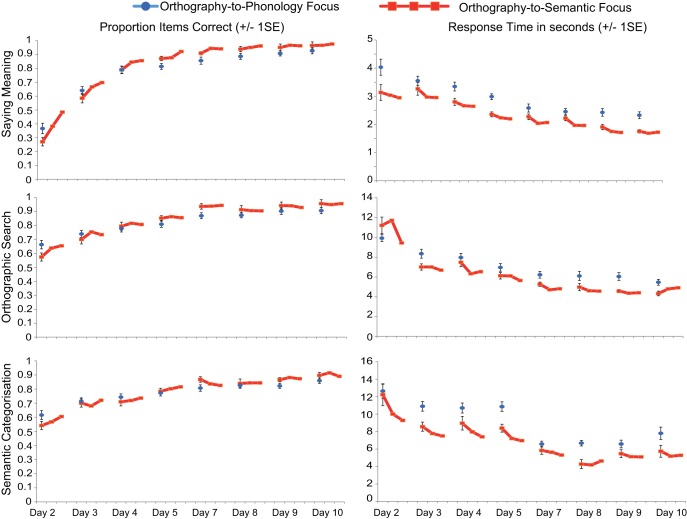
Accuracy and RTs in tasks that involved mapping between orthography and semantics for the O–P and O–S focus languages on each day and each session of training. Error bars are shown only for the first session on each day because only these data points were statistically compared. See the online article for the color version of this figure.

In order to assess the impact of the learning focus manipulation, we conducted ANOVAs comparing accuracy and RTs for the two languages in the first session of each of the 8 training days, on all six training tasks. Tasks that emphasized mappings between orthography and phonology (O–P tasks) are shown in Figure 3 and tasks that emphasized mappings between orthography and semantics (O–S tasks) are shown in Figure 4. Missing data, for example due to computer error, or if RT data were missing because a participant gave no correct responses in a particular condition, were replaced with the mean for that day and language. In these and all subsequent analyses, where Mauchley’s test indicated that the assumption of sphericity was violated, degrees of freedom were adjusted using the Greenhouse-Geisser correction.[Fig-anchor fig3][Fig-anchor fig4]

The results of all ANOVAs are presented in Table 1. Performance (accuracy and RT) improved across the training days for all tasks. For the three O–P tasks (reading aloud, spelling, rhyme judgment), accuracy was higher and RTs were faster for the O–P than the O–S focus language, although for accuracy this effect reduced as performance reached ceiling toward the end of training. In contrast, for the three O–S tasks (saying meanings, orthographic search, semantic categorization), accuracy did not differ between the two languages, except on the first training day, when it was higher for the O–P focus language, and on the last training day, when it was higher for the O–S focus language. RTs for the O–S tasks were faster throughout training for the O–S focus language.[Table-anchor tbl1]

Overall, training that focused on orthography-to-phonology mappings was more beneficial than training that focused on orthography-to-semantic mappings; accuracy was higher and responses were faster in tasks that required mapping between print and sound, and accuracy was equivalent in tasks that required mapping between print and meaning. The only benefit from training that focused on orthography-to-semantic mappings was increased speed in mapping between print and meaning.

#### Transfer between orthography–phonology and orthography–semantic training

**Figure 5 fig5:**
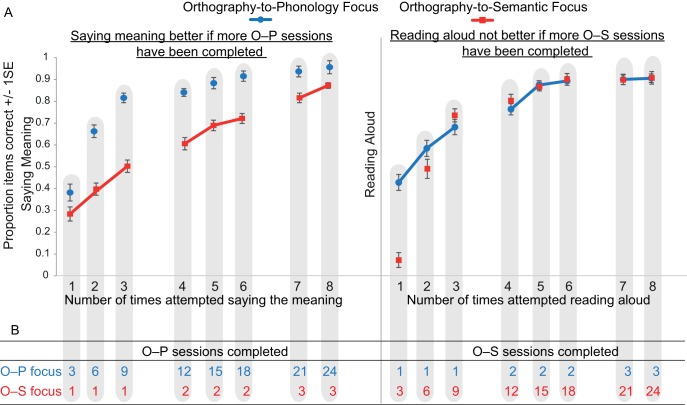
Replotting of data shown in the top left graphs of Figures 3 and 4 to illustrate how having completed relatively more O–P training sessions benefits saying the meanings (left panel), whereas having completed relatively more O–S training sessions does not benefit reading aloud (right panel). Graphs show the proportion of items correct in saying the meaning (left panel) and in reading aloud (right panel) the first eight times (after the initial scanning session) participants completed these tasks for the O–P and O–S focus languages. Joined up points indicate that sessions were completed on the same day, whereas separated points indicate that sessions were completed on different days. The information in the table that is connected to the graphs by gray lozenges shows how, when the number of times saying the meaning is equated, participants have received relatively more O–P training for the O–P than the O–S focus language (left panel). Conversely, when the number of times reading aloud is equated, participants have received relatively more O–S training for the O–S than the O–P focus language (right panel). See the online article for the color version of this figure.

The previous analyses established that tasks that required print-to-sound mapping were more accurate when training focused on O–P than O–S mappings. In contrast, accuracy was equivalent on tasks that required print-to-meaning mapping whether training primarily focused on O–P or O–S mappings. This suggests that knowledge of O–P mappings transferred and benefited access to item meanings as well as item sounds. To provide further evidence for this observation, we compared learning trajectories in saying the meanings for the two languages when the number of times participants had attempted this particular task was equated, but when the amount of orthography–phonology focused training they had completed was much greater for the O–P than the O–S focus language. We also compared learning trajectories for the two languages when the number of times participants had attempted reading aloud was equated, but when the amount of orthography–semantic focused training was much greater for the O–S than the O–P focus language. These comparisons are displayed in Figure 5.[Fig-anchor fig5]

In order to assess the impact of transfer quantitatively, we conducted ANOVAs on the proportion of items correct the first eight times (factor = session) participants said the meanings versus read aloud (factor = task) items from the O–P versus the O–S focus language (factor = training focus). This meant that for saying the meanings, sessions for the O–P focus language came from each of the 8 days of behavioral training, whereas sessions for the O–S focus language came from Days 2 to 4. In contrast, for reading aloud, sessions for the O–S focus language came from each of the eight days of training whereas sessions for the O–P focus language came from Days 2 to 4.^3^ As set out in Figure 5B, this provided us with imbalances in the type of training received, which enabled us to examine how additional O–P versus O–S focused training impacted on saying the meanings and reading aloud, respectively.

The ANOVA revealed that overall accuracy was higher for saying the meanings than reading aloud, *F*(1, 23) = 7.08, *p* = .01, η^2^ = .24, and for the O–P than the O–S focus language, *F*(1, 23) = 30.88, *p* < .001, η^2^ = .57. These effects were qualified by a significant interaction between task and training focus, *F*(1, 23) = 14.58, *p* = .001. η^2^ = .39. Participants were significantly more accurate at saying the meanings for the O–P focus than the O–S focus language, whereas accuracy in reading aloud was equivalent for the two languages. Planned comparisons demonstrated that the advantage of the O–P over the O–S focus language in saying the meanings was present in all sessions, but greater in magnitude in earlier sessions, and that there was no advantage for the O–S than the O–P language in reading aloud in any of the sessions. These analyses suggest that additional print-to-sound focused training boosted performance in saying the meanings, but that additional print-to-meaning focused training did not boost reading aloud.

### Performance During Test Sessions

We assessed participants’ performance in a number of ways on Days 6 (middle of training) and 12 (end of training). However, because the Day 6 data represent just a snap-shot of the training data reported in Figures 3 and 4, they are provided in the supplementary materials only.

#### Reading aloud and saying meanings of trained items (Figure 6A, Table 2)

**Figure 6 fig6:**
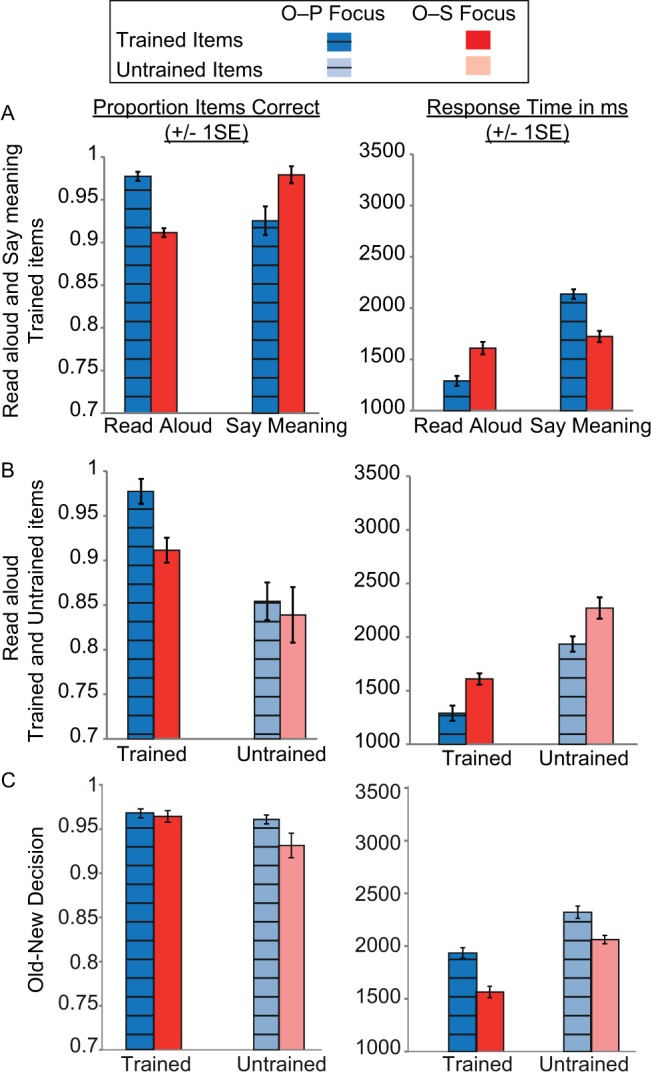
Accuracy and RTs in test tasks conducted at the end of behavioral training. (A) reading aloud and saying the meanings of trained items, (B) reading aloud trained and untrained items, (C) old-new decision. See the online article for the color version of this figure.

By the end of training, participants were able to read aloud and say the meanings of more than 90% of the items in both languages. Response times were around 1,500 ms. ANOVAs on both accuracy and RTs with the factors task (read aloud vs. say meaning) and training focus (O–P vs. O–S focus language) obtained significant interactions between these two factors; reading aloud was faster and more accurate for the language that received O–P than O–S focused training, but saying the meaning was faster and more accurate for the language that received O–S than O–P focused training (see Table 1). Thus, by the end of training, performance for each language was best for the task that had received most training.[Fig-anchor fig6][Table-anchor tbl2]

#### Generalization to untrained items (Figure 6B, Table 2)

The proportion of untrained items read aloud correctly from each language was around 0.8 with response times of 2,000 ms. An ANOVA on accuracy revealed an effect of training status (trained > untrained items), but no effect of language or interaction between training and language. For RTs, trained items were read aloud faster than untrained items and responses were faster for the O–P than the O–S language. There was no interaction between training status and language. The results confirm that untrained items were harder to read aloud than trained items. Furthermore, O–P focused training benefited reading aloud speed for both trained and untrained items relative to O–S focused training.

#### Old-new decision (Figure 6C, Table 2)

Discrimination between trained and untrained items was highly accurate; one-sample *t* tests on *d*-prime values indicated that performance was above chance for both the O–P focus language (*d*′ = 3.58) and the O–S focus language (*d*′ = 3.43) at the end of training. Paired *t* tests on *d*-prime values also indicated that discrimination accuracy did not differ for the two languages. An ANOVA showed that RTs were faster for trained than untrained items, and for the O–S than the O–P focus language. There was no interaction between training status and language.

#### Summary of test performance at the end of training

Overall, O–P focused training conferred benefits on reading aloud of trained and untrained items. In contrast, O–S focused training benefited saying item meanings, and also the speed with which participants could discriminate trained from untrained items.

### Functional MRI Data Prior to Behavioral Training

#### Reading aloud English words and pseudowords

Examining activity during English word and pseudoword reading allowed us to delineate the neural systems our participants used for reading aloud in their native language. In particular, we used the contrast pseudowords > words, because this should reveal dorsal stream brain regions involved in spelling-to-sound conversion and phonological output, and the contrast words > pseudowords, since this should reveal ventral stream brain regions involved in lexical and/or semantic processing (Taylor et al., 2013).

**Figure 7 fig7:**
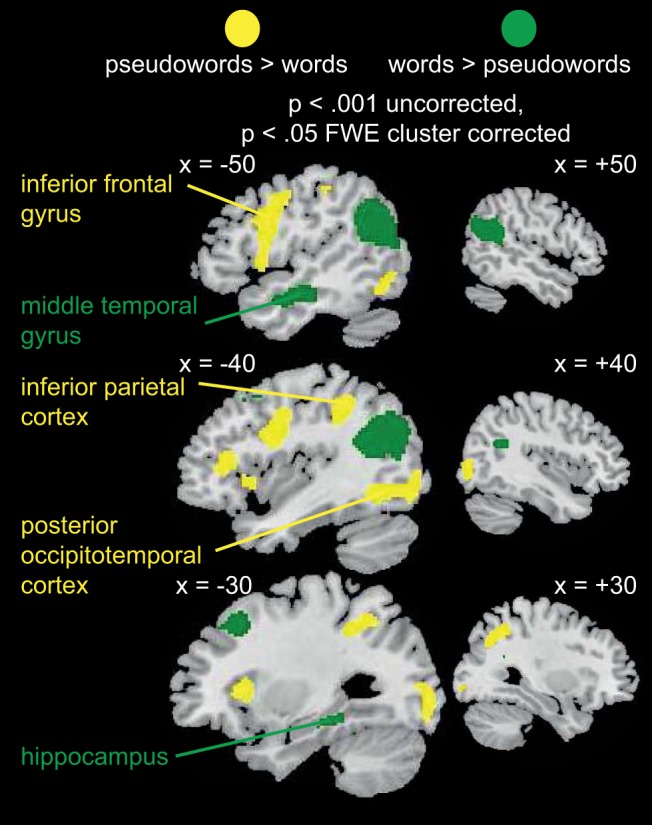
Brain regions that were more active during English pseudoword than word reading (yellow), or word than pseudoword reading (green). Left and right hemisphere slices show whole-brain activations at *p* < .001 voxelwise uncorrected and *p* < .05 FWE cluster-corrected for 20 participants.

Analyses were conducted on data from 20 participants, for whom the mean proportion of pseudowords and words read correctly is reported in Appendix D. Also provided in Appendix D are details of which subjects were excluded from each of the fMRI analyses, along with exclusion criteria. Note that we later compare English word and pseudoword reading with reading aloud the artificial orthographies. Therefore, as detailed in Appendix D, three participants were excluded from the current analysis because they performed poorly or moved excessively when reading aloud artificial orthographies at the end of training. We modeled errors, correct words, and correct pseudowords, and conducted paired *t* tests on pseudowords > words, and words > pseudowords. As shown in Figure 7 and Appendix E, pseudowords activated left inferior frontal and precentral gyri and the insula, bilateral inferior and superior parietal cortices, bilateral occipitotemporal cortices, supplementary motor area, and the right insula more than words. In contrast, words activated bilateral angular and supramarginal gyri, left middle temporal gyrus, precuneus, left middle frontal gyrus, and left hippocampus more than pseudowords. These results are very similar to those from a recent meta-analysis of neuroimaging studies, confirming that the phonologically mediated dorsal pathway is more active for pseudoword than word reading (Taylor et al., 2013) whereas the direct ventral pathway is more active for word than pseudoword reading.[Fig-anchor fig7]

#### Learning the pronunciations and meanings of novel words in the artificial orthographies

Participants learned pronunciations for the 24 trained items from the O–P focus language and meanings for the 24 trained items from the O–S focus language, while neural activity was measured with fMRI. Analyses were conducted on 18 participants, for whom the proportion of items recalled correctly during scanning is reported in Appendix D.

**Figure 8 fig8:**
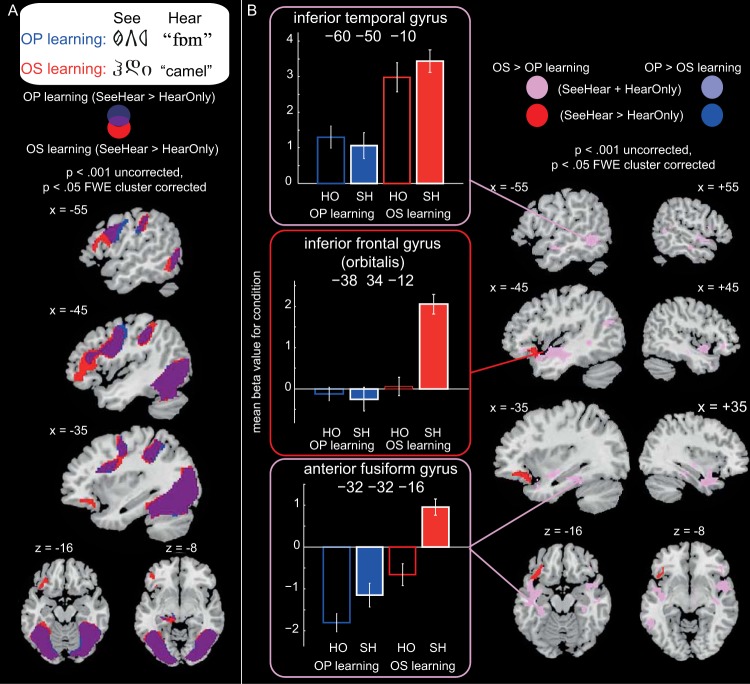
Brain regions active when learning pronunciations (OP learning) and meanings (OS learning) of artificial orthographies in MRI Scan 1, prior to behavioral training. Left and right hemisphere slices show whole-brain activations at *p* < .001 voxelwise uncorrected and *p* < .05 FWE cluster-corrected for 18 participants. Panel A shows the simple effect of see-hear > hear-only activity for OP (blue) and OS (red) learning, and the overlap between these (purple). Panel B shows the results of an ANOVA that included the factors learning type (OP vs. OS) and trial type (see-hear vs. hear-only). Slices show the main effect of learning type (pink: OS > OP, light blue: OP > OS) and the interaction between learning type and trial type that was driven by greater activation for see-hear relative to hear-only trials for OS than OP learning (red). Note that no brain regions showed more activation for OP than OS learning. Plots show activation for hear-only (HO) and see-hear (SH) trials for OP and OS learning at representative peak voxels that showed a main effect of learning type (pink boxes) or an interaction between learning and trial type (red box).

We modeled four event types in each O–P and O–S learning run: hear-only, see-hear, see-think, and see-speak. To examine the brain regions involved in *learning* pronunciations (O–P) and meanings (O–S) of novel words written in artificial orthographies, we conducted an ANOVA with two factors, learning type (O–P vs. O–S) and trial type (see-hear vs. hear-only), collapsed across the two runs. The same spoken forms were presented on see-hear and hear-only trials, but only see-hear trials afforded an associative learning opportunity. Therefore, the main effect of trial type, in particular the directional contrast (see-hear > hear-only), should reveal brain regions involved in learning the artificial orthographies. The main effect of learning type will reveal differences in activity between O–P and O–S learning. However, because this may have partly been driven by differences in listening to meaningful versus nonmeaningful spoken words, we then looked for an interaction between learning and trial type. In particular, for differences between O–P and O–S learning that were greater for see-hear than hear-only trials. Results are shown in Figure 8 and peak coordinates are reported in Appendix F.[Fig-anchor fig8]

The main effect of trial type revealed that there was greater activation for see-hear than hear-only trials in bilateral occipitotemporal cortices, thalamus, and bilateral inferior frontal and precentral gyri. To provide additional information, rather than showing the main effect, Figure 8A shows the simple effect of see-hear > hear-only activity for O–P and O–S learning and the overlap between them. This analysis demonstrates that both dorsal and ventral processing streams of the reading network are involved in learning pronunciations and meanings of artificial orthographies. There was also a main effect of learning type (Figure 8B); activation was greater for O–S than O–P learning in left fusiform and parahippocampal gyri, left temporal pole and inferior frontal gyrus (orbitalis), right temporal pole and inferior frontal gyrus (orbitalis), bilateral superior and medial frontal gyri, left inferior and middle temporal gyri, and the cerebellum. O–P learning did not activate any brain regions more than O–S learning. Finally, an interaction between learning type and trial type was obtained in left inferior frontal gyrus (orbitalis). As can be seen in the plots in Figure 8B, this interaction was driven by greater see-hear relative to hear-only activation for O–S than O–P learning. The plots also show that, although a significant interaction was not obtained in left anterior fusiform, this region showed a very similar profile to left inferior frontal gyrus (orbitalis), with only the see-hear O–S learning trials showing positive activation relative to rest. These analyses demonstrate that learning the arbitrary (English) meanings of novel words written in an artificial orthography activates ventral stream regions of the reading network previously implicated in semantic processing (Binder, Desai, Graves, & Conant, 2009) including both left inferior frontal gyrus (orbitalis) and left anterior fusiform, more than is the case for learning the systematic pronunciations of novel words.

**Figure 9 fig9:**
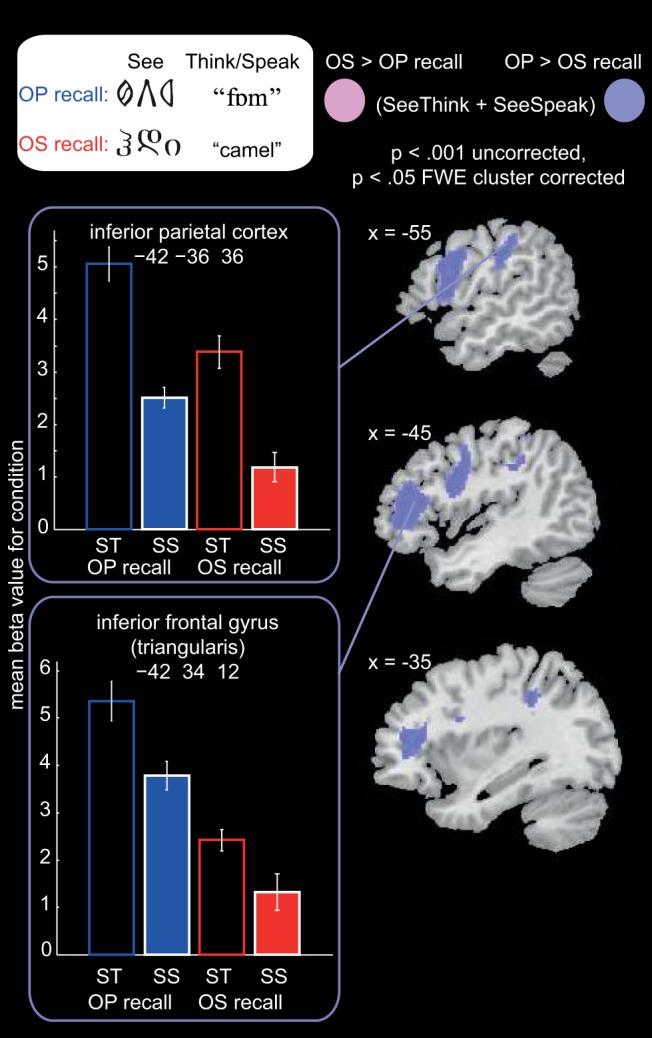
Brain regions showing differential activity when recalling pronunciations (OP recall) versus meanings (OS recall) of artificial orthographies, in MRI Scan 1, prior to behavioral training. Left hemisphere slices show the results of paired *t* tests between these two tasks (pink: OS > OP, light blue: OP > OS), collapsed across see-think (ST) and see-speak (SS) trials. Note that no brain regions showed greater activity when recalling meanings than pronunciations. Whole-brain activations are presented at *p* < .001 voxelwise uncorrected and *p* < .05 FWE cluster-corrected for 18 participants. Plots show activation for see-think and see-speak trials for OP and OS recall at representative peak voxels that showed greater activity for recalling pronunciations (OP) than meanings (OS).

Next we examined differential activation during *recall* of pronunciations versus meanings. Two paired *t* tests, O–P > O–S, and O–S > O–P, were conducted, collapsed across the two runs, and collapsed across see-think and see-speak trials. See-speak trials always immediately succeeded see-think trials for the same item and so the demands on recalling (pronunciation or meaning) were greatest during see-think trials. However, both see-think and see-speak trials were included in this analysis because, at this early stage of learning, participants were likely to still be partially retrieving pronunciations during see-speak trials. This assertion is supported by the fact that mean response times for reading aloud and saying the meanings were between 3,000 ms and 4,500 ms on the first day of behavioral training, and the time between the onset of see-think and see-speak trials was 3500ms. Results are shown in Figure 9 and peak coordinates are reported in Appendix G. Activation was greater during pronunciation (O–P) than meaning (O–S) recall in left inferior frontal gyrus (triangularis and opercularis) and left precentral gyrus, cerebellum, and left inferior parietal cortex and postcentral gyrus. The reverse contrast, O–S > O–P recall, did not reveal any significant clusters. The plots in Figure 9 show activation when retrieving O–P versus O–S associations during both see-think and see-speak trials. These analyses suggest that recalling systematic pronunciations of novel orthographic forms activates dorsal stream regions of the reading network, including left inferior frontal, motor, and inferior parietal cortices, more than recalling arbitrary meanings of orthographic forms.[Fig-anchor fig9]

### Functional MRI Data After the End of Training

#### Reading aloud trained and untrained artificial orthography items

Participants read aloud the 24 trained items from the O–P and the O–S focus languages, as well as 24 untrained items from each language, while neural activity was measured with fMRI. Twenty participants were included in the analyses, for whom the proportion of trained and untrained items read aloud correctly in each language during scanning is reported in Appendix D.

**Figure 10 fig10:**
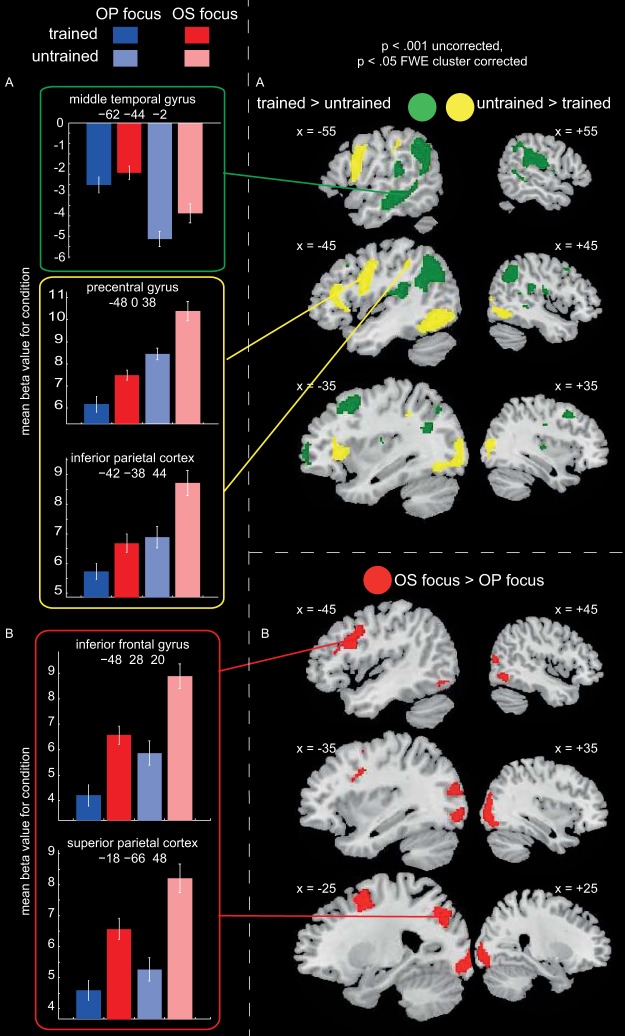
Brain activity during reading aloud in MRI Scan 2 at the end of training. Slices show main effects from an ANOVA that included the factors lexicality (trained vs. untrained) and training focus (OP vs. OS), which was conducted on activation during see-think trials, for items that were read aloud correctly. Whole-brain activations are presented at *p* < .001 voxelwise uncorrected and *p* < .05 FWE cluster-corrected for 20 participants. (A) Slices show main effect of lexicality, green = trained > untrained, yellow = untrained > trained. (B) Slices show main effect of training focus, red = OS > OP focus. Note that no brain regions showed more activation for the OP than the OS focus language. Plots for A and B show see-think activation for trained (dark bars) and untrained items (light bars) for the OP (blue bars) and the OS (red bars) focus languages, at representative peak voxels that showed greater activity for trained than untrained items (green box), untrained than trained items (yellow box), or the OS relative to the OP focus language (red box).

Sixteen event types were defined according to the following factors: O–P or O–S focus language, trained or untrained item, see-think or see-speak trial, correct or incorrect response on corresponding see-speak trial for that item. Incorrect trials were excluded from the imaging analyses to ensure that any differences in activity did not reflect in-scanner differences in performance between conditions. We only analyzed see-think trials as, at this late stage of training, there should have been enough time for participants to retrieve pronunciations during see-think trials, with the see-speak trial for the same item that immediately followed only requiring overt articulation. This assertion is supported by the fact that mean response times at the end of training were under 2,500 ms for reading aloud both trained and untrained items (as well as saying the meanings of trained items), and the time between the onset of see-think trials and see-speak trials was 3,500 ms. We conducted an ANOVA on see-think trials with the factors lexicality (trained vs. untrained) and training focus (O–P vs. O–S focus language). The main effect of lexicality should reveal how familiarity with an item’s whole word phonological and orthographic form, as well as knowledge of its meaning (English translation), influenced activity during reading aloud. The main effect of training focus should reveal how activity during reading aloud is influenced by training that has focused on orthography-to-semantic versus orthography-to-phonology associations. Results are shown in Figure 10 and peak coordinates are reported in Appendix H.[Fig-anchor fig10]

There was a main effect of lexicality (Figure 10A). Untrained relative to trained items activated left precentral and inferior frontal gyri, supplementary motor cortex, bilateral inferior and middle occipital cortices, and left inferior parietal cortex. In contrast, activity was greater for trained relative to untrained items in bilateral angular and middle temporal gyri, extending into supramarginal gyri, bilateral middle and superior frontal gyri, and the cuneus. Thus, it appears that reading untrained items in an artificial orthography activates similar dorsal pathway frontal, parietal, and occipital regions as reading English pseudowords. In contrast, reading trained items activates similar temporal lobe regions to reading English words.

There was also a main effect of training focus (Figure 10, Panel B). While no brain regions showed greater activity for the O–P than the O–S focus language, bilateral occipitotemporal cortices, left superior parietal cortex, and left precentral and inferior frontal gyri were more active for the O–S than the O–P focus language. Reading aloud was slower and more error prone for the O–S than the O–P focus language throughout training. Similarly, reading pseudowords is slower and more error prone than reading words. Therefore, effort in reading aloud appears to modulate activity in similar dorsal pathway brain regions for both artificial orthographies and English words. No brain regions showed a significant interaction between lexicality and training focus.

#### Similarity in activation patterns for artificial orthographies and English words

As discussed in the preceding section, comparing Figures 7 and 10 suggests that reading aloud untrained relative to trained artificial items (collapsed across both languages) activates similar brain regions to reading aloud pseudowords relative to words in English. To quantify the similarity in the brain responses obtained from these contrasts we computed the Fisher Z-transformed correlation between the SPM T-maps for the contrasts untrained > trained items, and pseudowords > words, for each subject. In other words, we examined whether voxels that showed greater activity for pseudowords than words, also showed greater activity for untrained than trained artificial orthography items, and vice versa. We constrained our analysis to gray matter voxels for each subject, and to voxels that were included in both the group analysis of the English words in MRI Scan 1, and the group analysis of the artificial items in MRI Scan 2. The mean correlation across subjects between activity for the untrained > trained and pseudowords > words contrasts was .32 (within subject standard error [*SE*] = .06), which was significantly greater than zero, *t*(19) = 5.64, *p* < .001. Thus, brain regions that were more active when reading aloud untrained than trained items were also more active when reading aloud English pseudowords relative to words.^4^

We then conducted a similar analysis to test the similarity between the brain responses obtained for the contrasts O–S > O–P focus, collapsed across trained and untrained items, and pseudowords > words, because both of these contrasts activated dorsal brain regions that are engaged when reading aloud is effortful. The mean correlation across subjects was .17 (*SE* = .04), which was significantly greater than zero, *t*(19) = 3.92, *p* < .001.^5^ Thus, training that focuses on orthography-to-semantic rather than orthography-to-phonology associations results in more effortful reading aloud, and a pattern of brain activity that resembles pseudoword more than word reading, for both trained and untrained items.

#### Saying the meanings of trained items

In the MRI scan the day after the final training session, participants also said the meanings for all the trained items from both the O–P and the O–S focus languages. Analyses were conducted on 20 participants, for whom the proportion of item meanings said correctly during scanning is reported in Appendix D. Eight event types were defined according to the following factors: O–P or O–S focus language, see-think or see-speak trial, correct or incorrect response on corresponding see-speak trial for that item. Incorrect trials were again excluded from the imaging analyses and only see-think trials were analyzed. A paired *t* test revealed no difference in see-think trial activity for saying the meanings of items in the O–P versus the O–S focus language, even at an uncorrected threshold of *p* < .001.

To ensure that differences between the two languages were not missed due to a lack of sensitivity, we conducted the same analysis within 10 mm spherical regions of interest (ROIs) based on peak coordinates from the English word reading contrasts (words > pseudowords; left angular gyrus −56, −54, 24; left anterior middle temporal gyrus −48, −6, −18 and pseudowords > words; left inferior frontal gyrus −42, 0, 28; left intraparietal sulcus: −20, −62, 48; left inferior temporal gyrus: −44, −60, −8, in the same participants. The same paired *t* test was nonsignificant, *t*(19) < 1, ns, in all five of these ROIs. We further used ROIs based on peak coordinates that showed activation differences between O–P and O–S learning or recall in Scan 1. Left anterior fusiform gyrus (−32, −32, −16) and left inferior frontal gyrus orbitalis (−38, 34, −12) were more active for learning O–S than O–P associations in Scan 1, but did not show differential activity for saying the meanings of items from the two languages in Scan 2 (both *t* < 1, ns). Similarly, left inferior frontal gyrus triangularis (−42, 34, 12) and left inferior parietal cortex (−42, −36, 36) were more active for recalling O–P than O–S associations in Scan 1, but did not show differential activation for saying the meanings of items from the two languages in Scan 2 (both *t* < 1, ns). These analyses demonstrate that brain activity was not modulated by whether training had focused on orthography-to-phonology or orthography-to-semantic mappings, when the task was to say the meanings of the trained words.

## Discussion

There is strong scientific consensus that reading instruction that focuses on the relationship between letters and sounds is beneficial for learning to read an alphabetic script (National Reading Panel, 2000; Rayner, Foorman, Perfetti, Pesetsky, & Seidenberg, 2001; Rose, 2006). However, the extent to which this practice is adopted in classrooms varies, from intensive phonic training to multicuing environments that combine phonic and meaning-related cues (e.g., predictions based on pictures or preceding context). Interpreted within the context of cognitive models, these differences in emphasis correspond to a focus on learning to read via the phonologically mediated print-to-sound-to-meaning pathway versus the direct print-to-meaning pathway. In this research, we sought to understand the behavioral and neural consequences of a relative difference in emphasis on learning via these two pathways.

To do so, we taught adults to read new words written in artificial alphabets, and compared two methods of instruction—one that focused on acquisition of print-to-sound associations, and another that focused on acquisition of print-to-meaning associations. Prior to this behavioral training, we demonstrated that these methods of instruction did indeed tap into distinct reading pathways by examining neural activity while participants were learning sound or meaning associations for the new written words. We evaluated the relative merits of these training regimes for both reading aloud and comprehension of written words in terms of behavioral performance at different points in training, and used brain imaging data collected at the end of training to help us to understand the mechanisms that underpinned these behavioral outcomes.

### Summary of Results and Implications

#### Print-to-sound training benefits learning to decode and comprehend

Participants learned to read the two languages over eight days. For one, they received three times as much training focused on associating print with sound than training focused on associating print with meaning, whereas for the other the reverse was true. Throughout training, reading aloud and spelling were more accurate and faster for the print-to-sound focused language than for the print-to-meaning focused language (see Figure 3). Additionally, generalization to reading aloud untrained items at the end of training, which required knowledge of individual letter sounds, was highly accurate for both languages, but faster for the print-to-sound focused language (Figure 6B). Conversely, throughout most of the training period, accuracy in saying the item meanings was equivalent for the two languages, but faster for the print-to-meaning focused language (see Figure 4). Thus, print-to-meaning training benefitted the speed, but not the accuracy, with which word meanings can be retrieved from their written forms.

The observation that accuracy in saying item meanings during training was equivalent for the two languages suggests that print-to-sound training conferred benefits for this task, as well as for reading aloud. To assess whether this was the case, we conducted an additional analysis that equated the number of times participants had attempted to say the word meanings in the two languages (i.e., once on each of the eight training days for the O–P focus language, and the first eight times across Days 2 to 4 for the O–S focus language). This revealed that accuracy in saying the meanings was far superior for the print-to-sound focused language than for the print-to-meaning focused language (left panel Figure 5). Thus, the additional print-to-sound training participants received for the O–P focus language across the 8 training days transferred and benefited comprehension of printed words. In contrast, when we conducted the equivalent analysis to equate the number of times participants had attempted to read aloud the words in the two languages, additional print-to-meaning training across the 8 training days did not confer any benefits to reading aloud (right panel Figure 5).

In summary, the benefits of the two forms of training were asymmetric. Print-to-sound training drove greater accuracy and speed in reading aloud as well as transferring and benefiting accuracy in comprehending printed words. Conversely, print-to-meaning training drove faster but not more accurate comprehension during training, and had no transferrable benefit to reading aloud. Furthermore, although performance in reading aloud and comprehension for the two languages converged toward the end of training, this is likely due to the limited number of words participants had to learn. If our languages, like natural alphabetic orthographies, comprised a limited set of letters but a virtually limitless number of words, we would expect the benefits of print-to-sound relative to print-to-meaning training to persist, because letter–sound mappings are systematic across words, but print–meaning mappings remain arbitrary. Overall, the asymmetric benefits of the two forms of training are consistent with the claim that using phonic-based methods is a better use of limited instructional time than using meaning-based methods, both for learning to read aloud and comprehend written words accurately (e.g., Rayner et al., 2001).

#### Learning print-to-sound and print-to-meaning associations engages the dorsal and ventral reading pathways, respectively

Our predictions about the neural consequences of an instructional focus on print-to-sound versus print-to-meaning mappings were predicated on an assumption that these forms of learning tapped into distinct reading pathways. To confirm that this was the case, prior to behavioral training, we measured neural activity while participants learned print-to-sound mappings for one language, and print-to-meaning associations for the other language. During training blocks we did indeed observe ventral pathway specialization for learning print-to-meaning associations, which activated left anterior fusiform and ventral inferior frontal gyrus more than learning print-to-sound associations (see Figure 8). Thus, our print-to-meaning task engaged ventral stream regions, despite concerns about the difficulty of imaging this area with conventional fMRI methods (Visser, Jefferies, & Lambon Ralph, 2010). Conversely, we observed dorsal pathway specialization during testing blocks—recalling print-to-sound associations activated left inferior parietal cortex and dorsal inferior frontal gyrus more than recalling print-to-meaning associations (see Figure 9). These results demonstrate the division of labor between acquiring systematic componential print-to-sound mappings (dorsal stream), and acquiring arbitrary holistic print-to-meaning mappings (ventral stream). However, future research will be necessary to determine why these effects were differentially observed during training and testing blocks.

#### Reading aloud artificial orthographies after training engages similar brain regions to reading aloud in English

One goal of this study was to use fMRI to reveal the neural consequences of particular forms of reading instruction. Because previous research has sometimes questioned the utility of artificial language approaches for making inferences about natural language processes (see Pothos, 2007) we sought to quantify the similarity between neural activity during reading aloud the artificial orthographies and during reading aloud English stimuli. For each participant, we compared the spatial distribution of activation during reading aloud of untrained relative to trained items (following 2-weeks of training), and during reading aloud of English pseudowords relative to words. This analysis demonstrated that voxels that were more active for pseudoword than word reading were also more active for untrained than trained items in the artificial orthography. Conversely, voxels that were more active for word than pseudoword reading were also more active for trained than untrained items in the artificial orthography. Thus, there was a striking (and statistically reliable) similarity between the patterns of brain activity evoked when reading the artificial orthographies and those evoked when reading English items. These data suggest that this laboratory paradigm has the potential to inform questions pertaining to reading instruction.

#### Print-to-meaning training increases the neural effort associated with reading aloud

To determine why reading aloud performance was worse following print-to-meaning than print-to-sound training, we examined how learning focus impacted on neural activity during reading aloud at the end of training. During trained and untrained item reading, dorsal pathway regions were more active for the print-to-meaning than for the print-to-sound focus language. Multivariate analyses further showed that voxels that were more active for reading aloud in the print-to-meaning than the print-to-sound focused language were also more active for English pseudoword than word reading. The fact that reading aloud the print-to-meaning focused language evoked a similar spatial distribution of activity to that obtained when reading aloud pseudowords suggests that phonologically mediated reading was more effortful for this than for the print-to-sound focused language (Taylor et al., 2013; Taylor, Rastle, & Davis, 2014b). Furthermore, this was not accompanied by changes in direct pathway use. That is, there was no difference in activity in ventral brain regions following the two training types. These data imply that in an alphabetic script, teaching that focuses on print-to-meaning rather than print-to-sound relationships may increase the neural effort of phonologically decoding written words, and that this does not appear to be compensated by alternative strategies.

#### Print-to-sound training does not increase the neural effort involved in comprehending written words

To examine why learning to comprehend the novel words was not worse for the print-to-sound than the print-to-meaning focus language, we also measured neural activity while participants said the meanings of trained items from both languages. We did not observe any differences in activation for the print-to-sound versus the print-to-meaning focused language in this task. This was the case even when we conducted more sensitive ROI analyses targeted at relevant regions, for example those in the ventral stream that were active when participants learned meanings for the novel words before training. Thus, focusing on print-to-sound versus print-to-meaning associations during learning did not change reliance on either the phonologically mediated dorsal pathway or the direct ventral pathway, when generating word meanings. These analyses imply that phonic-based teaching methods should not increase the neural effort involved in comprehending printed words relative to meaning-based teaching methods.

### Outstanding Issues

#### Would print-to-meaning training be more beneficial for inconsistent words?

Triangle model simulations (Harm & Seidenberg, 2004; Plaut et al., 1996) and empirical data (Taylor, Duff, Woollams, Monaghan, & Ricketts, 2015) demonstrate that semantic knowledge facilitates reading aloud for inconsistent more than consistent words. Might print-to-meaning training, therefore, be more beneficial for learning to read novel words with inconsistent spelling-to-sound mappings? With respect to learning to read aloud, print-to-meaning training might benefit inconsistent more than consistent words. This is because learning print-to-sound mappings is more difficult for the former, and they might therefore benefit from support from the print-to-meaning-to-sound pathway. However, this does not imply that print-to-meaning training would be more beneficial than print-to-sound training for learning to read aloud inconsistent words. In fact, this seems extremely unlikely, because even for inconsistent words the relationship between print and sound is relatively systematic which aids learning, whereas the relationship between print and meaning is arbitrary and very difficult to learn. In summary, for reading aloud we would still expect print-to-sound training to be more beneficial than print-to-meaning training for inconsistent words.

Considering learning to comprehend, triangle model simulations show that the orthography–semantic pathway is equally accurate at learning to generate meaning from print for consistent and inconsistent words. This is because these mappings are arbitrary, and therefore difficult to learn, for both item types. It therefore seems likely that print-to-sound training would be more beneficial than print-to-meaning training for learning to comprehend both consistent and inconsistent words. This is because it enables learners to capitalize on print-to-sound systematicities (which are present even for inconsistent words), and then use their preexisting oral vocabulary knowledge to map from sound to meaning.

Overall, for both learning to read aloud and comprehend written words, reading instruction should focus on the systematicities that are present in a writing system. For alphabetic scripts, this means teaching the systematicities that exist in print-to-sound mappings for both consistent and inconsistent words, not teaching arbitrary print-to-meaning mappings, which will be difficult to learn for all words.

#### How does the way children learn to read differ from adults in the current study?

Despite the neural similarity between reading our artificial scripts and reading English stimuli, there are many features of our experiment that are unlike children learning to read for the first time. One key feature of the current study was that each participant learned to read two different orthographies simultaneously. Though we tried to minimize any interference between the two languages by using different vowel sounds, visually distinct scripts, and different sets of meanings for the two languages, we recognize that this design choice may have increased the difficulty of the task. Participants in our experiment also learned to associate the phonological and orthographic forms of the new languages with familiar meanings, for which they already possess English spoken and written word form representations. This decision was partly taken for pragmatic reasons, since it would have increased the difficulty of the learning task if participants had to learn novel meanings (see, e.g., Taylor, Plunkett, & Nation, 2011). In addition, novel meanings would have been weakly represented, whereas in one’s native language the words we learn to read early on are usually highly familiar oral vocabulary items. Nonetheless, possessing alternative (English) orthographic and phonological forms for the items made the learning task different from that facing children learning to read in their native language.^6^

More generally, it could be argued that the adult participants in our study came to the artificial language tasks with a fully developed reading system in place, which may have influenced their approach to the tasks and their neural responses to the novel words. Indeed, our participants were already aware that letters correspond to sounds, and this likely helped with the print-to-sound focused tasks and the extraction of phonic knowledge. However, the reading aloud learning curves (see Figure 3) demonstrate that symbol-to-sound learning was nontrivial. Furthermore, had participants been able to rely on their preexisting reading system to learn the novel materials, we may have expected more pronounced use of the ventral pathway, because this is the primary system used by skilled readers (Cohen & Dehaene, 2009). Instead, our results suggested that even when whole-word meaning information was emphasized during training (i.e., for the O–S focused language), participants primarily accomplished our learning tasks via the dorsal pathway. This is exactly as we would expect children to approach the task, based on the hypothesis of a dorsal-to-ventral shift in reading acquisition (Pugh et al., 2000; Rueckl & Seidenberg, 2009; Sandak et al., 2012). Nonetheless, artificial language learning studies should be viewed as only one piece of evidence, complementary to more naturalistic studies of children’s development, to solve the problem of reading acquisition.

## Conclusions

Our study capitalized on the experimental control provided by artificial language learning methods to quantify the relative benefits of print-to-sound versus print-to-meaning focused training for learning to read aloud and comprehend single written words. In our experiment, as for children learning to read alphabetic languages, oral vocabulary was pretrained and print–sound mappings were systematic. Under these circumstances, the benefits of print-to-sound, relative to print-to-meaning, training were striking and can be summarized as follows: (a) reading aloud trained words was faster and more accurate, (b) generalization in reading aloud untrained words was faster, and (c) comprehension of written words was more accurate earlier in learning. These findings therefore provide experimental support for the importance of phonics instruction in early years teaching. In particular, our findings contradict the suggestion that phonics teaching does not aid learning to read for meaning (e.g., Davis, 2013).

Brain imaging data revealed considerable overlap between neural activity when reading aloud the artificial languages and when reading English words and pseudowords. This increases our confidence that data from artificial languages can provide insight into the cognitive and neural systems that contribute to natural language learning. Given this overlap, a crucial finding was that activity in the phonologically mediated dorsal pathway during reading aloud was greater following print-to-meaning than print-to-sound training. This likely reflects increased effort in mapping from spelling to sound. Furthermore, this neural disadvantage was not compensated by increased engagement of, or reduced effort in, the direct ventral pathway, either during reading aloud or reading comprehension. These data therefore imply that learning that focuses on arbitrary associations between print and meaning may not promote use of direct print-to-meaning associations, and instead hinders use of print-to-sound relationships. These print-to-sound relationships have been shown by our work to be crucial not only for successful reading aloud but also for accurate written word comprehension.

In sum, this experiment investigated the behavioral and neural consequences of different methods of reading instruction for learning to read single words in alphabetic writing systems, in the case where oral vocabulary is relatively secure. Under these circumstances, our findings suggest that interventions aiming to improve the *accuracy of reading aloud and/or comprehension* in the early stages of learning should focus on the systematicities present in print-to-sound relationships, rather than attempting to teach direct access to the meanings of whole written words. Alongside broader oral language teaching, this means embracing phonics-based methods of reading instruction, and rejecting multicuing or balanced literacy approaches which, our results suggest, may hinder the discovery of spelling–sound relationships essential for reading aloud and comprehension.

## Supplementary Material

10.1037/xge0000301.supp

## Figures and Tables

**Table 1 tbl1:** Results of ANOVAs Assessing the Effect of Training Focus and Day on Accuracy and Response Times in the Six Training Tasks Across the 8 Days of Training

	Task	Main effect focus	Main effect day	Interaction
Accuracy in O–P tasks	Reading aloud	*F*(1, 23) = 30.81, *p* < .001, η^2^ = .57	*F*(2.13, 48.96) = 145.66, *p* < .001, η^2^ = .86	*F*(1.98, 45.58) = 11.07, *p* < .001, η^2^ = .33
	Spelling	*F*(1, 23) = 19.12, *p* < .001, η^2^ = .45	*F*(2.83, 5.14) = 64.01, *p* < .001, η^2^ = .73	*F*(2.97, 68.35) = 19.72, *p* < .001, η^2^ = .46
	Rhyme judgement	*F*(1, 23) = 7.72, *p* = .01, η^2^ = .25	*F*(1.93, 4.42) = 4.02, *p* < .05, η^2^ = .15	*F*(2.79, 64.24) = 3.30, *p* < .05, η^2^ = .13
				
Accuracy in O–S tasks	Say meaning	*F*(1, 23) < 1, ns	*F*(2.71, 62.24) = 109.48, *p* < .05, η^2^ = .83	*F*(3.27, 75.14) = 4.39, *p* < .01, η^2^ = .16
	Orthographic search	*F*(1, 23) < 1, ns	*F*(2.51, 57.68) = 26.08, *p* < .001, η^2^ = .53	*F*(3.41, 78.41) = 3.30, *p* < .05, η^2^ = .13
	Semantic categorization	*F*(1, 23) < 1, ns	*F*(3.16, 72.66) = 27.37, *p* < .001, η^2^ = .54	*F*(3.91, 89.98) = 2.50, *p* = .05, η^2^ = .10
				
RT in O–P Tasks	Reading aloud	*F*(1, 23) = 32.72, *p* < .001, η^2^ = .59	*F*(3.26, 75.05) = 99.51, *p* < .001, η^2^ = .81	*F*(2.70, 62.08) < 1, ns
	Spelling	*F*(1, 23) = 32.49, *p* < .001, η^2^ = .59	*F*(2.10, 48.36) = 49.34, *p* < .001, η^2^ = .68	*F*(2.77, 63.88) = 1.91, ns
	Rhyme judgement	*F*(1, 23) = 4.12, *p* = .05, η^2^ = .15	*F*(2.87, 66.02) = 65.24, *p* < .001, η^2^ = .74	*F*(2.23, 51.35) = 3.46, *p* < .05, η^2^ = .13
				
RT in O–S Tasks	Say meaning	*F*(1, 23) = 21.41, *p* < .001, η^2^ = .48	*F*(2.64, 60.76) = 37.27, *p* < .001, η^2^ = .62	*F*(3.39, 77.70) = 1.70, ns
	Orthographic search	*F*(1, 23) = 12.26, *p* < .01, η^2^ = .35	*F*(3.60, 82.83) = 29.44, *p* < .001, η^2^ = .56	*F*(3.99, 91.86) = 2.34, *p* = .06
	Semantic categorization	*F*(1, 23) = 15.61, *p* = .001, η^2^ = .40	*F*(2.54, 58.46) = 20.92, *p* < .001, η^2^ = .48	*F*(4.04, 92.82) = 1.72, ns

**Table 2 tbl2:** Results of Analyses Conducted at the End of Training That Assessed the Effect of Training Focus (O–P vs. O–S) on Accuracy and Response Times in Reading Aloud Versus Saying the Meanings of Trained Items, Reading Aloud Trained Versus Untrained Items, and Old-New Decisions to Trained Versus Untrained Items

Task	Main effect task	Main effect focus	Interaction
Reading aloud and saying the meaning of trained items			
Accuracy	*F*(1, 23) < 1, ns	*F*(1, 23) < 1 ns	*F*(1, 23) = 7.35, *p* = .01, η^2^ = .24
RT	*F*(1, 23) = 60.88, *p* < .001, η^2^ = .73	*F*(1, 23) < 1, ns	*F*(1, 23) = 31.10, *p* < .001, η^2^ = .58
Reading aloud trained and untrained items			
Accuracy	*F*(1, 23) = 21.55, *p* < .001, η^2^ = .48	*F*(1, 23) = 1.57, ns	*F*(1, 23) = 1.72, ns
RT	*F*(1, 23) = 57.31, *p* < .001, η^2^ = .71	*F*(1, 23) = 9.61, *p* < .01, η^2^ = .30	*F*(1, 23) < 1, ns
Old-new decisions to trained and untrained items			
D-Prime		*t*(23) = 1.20, ns	
RT	*F*(1, 23) = 66.23, *p* < .001, η^2^ = .74	*F*(1, 23) = 17.30, *p* < .001, η^2^ = .43	*F*(1, 23) = 1.90, ns

## Footnotes

1 The role of the left angular gyrus is somewhat difficult to determine, in part because it is typically deactive during reading relative to rest. Some have suggested that the reduced deactivation seen during word relative to pseudoword reading reflects semantic processing (Binder et al., 2009) as words may detract less from a “semantic resting state” (Binder, 2012). However, others challenge this on the basis that aphasic patients with angular gyrus lesions do not appear to have impoverished semantic representations, but rather problems using semantic, as well as nonsemantic, information flexibly (Humphreys & Lambon Ralph, 2015; Jefferies & Lambon Ralph, 2006).

2 The mean changes in proportion of items correct were as follows: Language 1 say meaning = −.01; Language 2 say meaning = −.01; Language 1 say novel spoken word = .01; Language 2 say novel spoken word = .06.

3 Note that participants additionally had two opportunities to read aloud words from the O–P focus language, and two opportunities to say the meanings of items from the O–S focus language, in the first scan that occurred before behavioral training.

4 Note that this was not driven by one language only: For the O–P focus language the correlation between the contrasts untrained > trained, and pseudoword > word was .27 (*SE* = .05), which was significantly greater than zero, *t*(19) = 5.34, *p* < .001, and for the O–S focus language the correlation was .24 (*SE* = .05), *t*(19) = 4.53, *p* < .001. There was no significant difference between the strength of these correlations, *t*(19) < 1.

5 This was also the case when trained and untrained items were analyzed separately; OS trained > OP trained correlation with pseudowords > words = .16 (*SE* = .04), *t*(19) = 3.35, *p* < .001; O–S untrained > O–P untrained correlation with pseudowords > words = .15 (*SE* = .04), *t*(19) = 4.03, *p* < .001. There was no significant difference between the strength of these correlations, *t*(19) < 1.

6 One reviewer suggested that participants may have learned to read the novel words by forming whole-word paired associates with their English forms (as in a translation task). Though this strategy is possible within our paradigm, participants’ ability to generalize (Figure 6B), as well as the impact of print-to-sound training on retrieving the meanings of novel words (see Figure 5), instead suggest that learning the systematic symbol-sound mappings was of primary importance, as it is for children learning to read alphabetic languages.

## A Item Set for Participant 1

**Table:**

Language 1			Language 2		
Trained	Generalization	Old-new untrained	Trained	Generalization	Old-new untrained
bəʊs	dəʊn	bəʊf	bæb	dæf	sæb
bəʊv	fəʊb	dəʊg	mæf	fæk	væd
dəʊf	məʊk	pəʊs	pæf	gæb	fæp
gəʊn	pəʊd	səʊg	væk	kæg	kæs
səʊm	vəʊn	təʊs	fæm	pæz	dæt
zəʊt	zəʊd	vəʊb	næv	tæm	bæv
faɪk	daɪt	daɪp	gid	biv	tig
maɪv	faɪm	faɪz	vid	kis	nik
maɪz	gaɪs	kaɪm	tip	pim	nim
naɪb	kaɪz	paɪm	zis	sif	gin
paɪg	naɪg	saɪv	kit	sig	zip
vaɪf	taɪf	zaɪn	diz	vib	dis
dεp	bεm	bεf	sɒn	dɒs	sɒf
gεd	bεz	gεn	gɒp	fɒd	zɒg
kεs	kεt	kεb	zɒs	mɒn	fɒm
tεp	mεp	mεk	mɒt	tɒn	pɒn
vεn	nεf	tεz	bɒv	vɒd	mɒv
zεk	sεv	zεt	dɒz	zɒt	bɒz
fʌb	gʌb	fʌd	kub	bup	gub
kʌg	pʌv	gʌk	tug	guz	tud
nʌz	sʌg	mʌp	nug	mup	kuf
pʌm	tʌs	nʌd	puk	nut	muk
sʌt	vʌp	nʌv	sum	nuv	put
tʌd	zʌk	vʌt	fun	zuk	vuz

## B Counterbalancing Details

**Table:**

Subject	Language 1						Language 2					
	Focus	Orthography	Item Set 1	Item Set 2	Item Set 3	Noun set	Focus	Orthography	Item Set 4	Item Set 5	Item Set 6	Noun set
1	O–P	Hungarian	Trained	Untrained	Old-new	1	O–S	Georgian	Trained	Untrained	Old-new	2
2	O–P	Hungarian	Untrained	Old-new	Trained	1	O–S	Georgian	Untrained	Old-new	Trained	2
3	O–P	Hungarian	Old-new	Trained	Untrained	1	O–S	Georgian	Old-new	Trained	Untrained	2
4	O–P	Hungarian	Trained	Untrained	Old-new	2	O–S	Georgian	Trained	Untrained	Old-new	1
5	O–P	Hungarian	Untrained	Old-new	Trained	2	O–S	Georgian	Untrained	Old-new	Trained	1
6	O–P	Hungarian	Old-new	Trained	Untrained	2	O–S	Georgian	Old-new	Trained	Untrained	1
7	O–S	Georgian	Trained	Untrained	Old-new	2	O–P	Hungarian	Trained	Untrained	Old-new	1
8	O–S	Georgian	Untrained	Old-new	Trained	2	O–P	Hungarian	Untrained	Old-new	Trained	1
9	O–S	Georgian	Old-new	Trained	Untrained	2	O–P	Hungarian	Old-new	Trained	Untrained	1
10	O–S	Georgian	Trained	Untrained	Old-new	1	O–P	Hungarian	Trained	Untrained	Old-new	2
11	O–S	Georgian	Untrained	Old-new	Trained	1	O–P	Hungarian	Untrained	Old-new	Trained	2
12	O–S	Georgian	Old-new	Trained	Untrained	1	O–P	Hungarian	Old-new	Trained	Untrained	2
13	O–P	Georgian	Trained	Untrained	Old-new	1	O–S	Hungarian	Trained	Untrained	Old-new	2
14	O–P	Georgian	Untrained	Old-new	Trained	1	O–S	Hungarian	Untrained	Old-new	Trained	2
15	O–P	Georgian	Old-new	Trained	Untrained	1	O–S	Hungarian	Old-new	Trained	Untrained	2
16	O–P	Georgian	Trained	Untrained	Old-new	2	O–S	Hungarian	Trained	Untrained	Old-new	1
17	O–P	Georgian	Untrained	Old-new	Trained	2	O–S	Hungarian	Untrained	Old-new	Trained	1
18	O–P	Georgian	Old-new	Trained	Untrained	2	O–S	Hungarian	Old-new	Trained	Untrained	1
19	O–S	Hungarian	Trained	Untrained	Old-new	2	O–P	Georgian	Trained	Untrained	Old-new	1
20	O–S	Hungarian	Untrained	Old-new	Trained	2	O–P	Georgian	Untrained	Old-new	Trained	1
21	O–S	Hungarian	Old-new	Trained	Untrained	2	O–P	Georgian	Old-new	Trained	Untrained	1
22	O–S	Hungarian	Trained	Untrained	Old-new	1	O–P	Georgian	Trained	Untrained	Old-new	2
23	O–S	Hungarian	Untrained	Old-new	Trained	1	O–P	Georgian	Untrained	Old-new	Trained	2
24	O–S	Hungarian	Old-new	Trained	Untrained	1	O–P	Georgian	Old-new	Trained	Untrained	2

## C Details of English Words and Nonwords Used in MRI Scan 1

**Table:**

Item type	Min letters	Max letters	Mean orthographic neighborhood	Mean log frequency	Mean imageability
Irreg High-Image	3	6	7.7	4.62	559.6
Irreg Low-Image	3	6	6.27	4.69	350.2
Reg High-Image	3	6	8.83	4.56	566.93
Reg Low-Image	3	6	8.83	4.60	351.83
Pseudoword	4	6	6.5	NA	NA

## D Individual Subject Performance and Exclusion Criteria in fMRI Tasks

**Table:**

	English words Scan 1		End of learning Scan 1		Scan 2					
Participant number	Words	Pseudowords	O–P	O–S	O–P read trained	O–P read untrained	O–S read trained	O–S read untrained	O–P say meaning	O–S say meaning
101	.98	.88	.79	.79	1.00	.92	1.00	.96	^a^	^a^
102	.95^d^	.87^d^	.04^b^	.25^bc^	.92^c^	.83^c^	.75^c^	.58^c^	.25^c^	.79^c^
103	.98	.95	.96	.96	.96	.96	1.00	.92	1.00	1.00
104	1.00	1.00	.92	.71	.96	.67	1.00	1.00	.96	1.00
105	^a^	^a^	.79	.75	.96^d^	.92^d^	.92^d^	.71^d^	.96	1.00
106	.98	.90	.17^b^	.25^b^	1.00	.96	.92	.88	.92	.92
107	.98	.98	.75	.79	.96	.96	1.00	.96	1.00	1.00
108	.98	.98	.75	.83	1.00	.92	.96	.58	.96	.96
109	.98	.97	.21^b^	.29^b^	1.00	.88	.92	.54	.83	.96
110	.99	.93	.21	.63	.88	.79	.83	.75	.50	.96
111	.99	.98	.46	.83	.96	.88	.96	.88	.96	.96
112	.97^d^	.97^d^	.25^b^	.33^b^	.7^b^	.46^b^	.42^b^	.04^b^	.42^b^	.71^b^
113	.96	.88	.21	.71	1.00	1.00	.29	.25	.79	.83
114	.99	.95	.29	.54	1.00	.96	.96	.96	.96	1.00
115	.99	.98	.88	.96	1.00	1.00	1.00	1.00	1.00	1.00
116	.97	.93	.67	.75	1.00	.54	1.00	.75	1.00	1.00
117	.99	.95	.50	.63	.96	.83	1.00	.88	.96	1.00
118	.96^d^	.80^d^	.21^b^	.46^b^	.42^b^	.46^b^	.42^b^	.46^b^	.38^b^	.83^b^
119	.97	.97	.21	.50	.88	.83	.96	.75	.92	1.00
120	.98	.95	.92	.63	1.00	1.00	.96	.92	.96	1.00
121	.98	.98	.63	.71	1.00	1.00	1.00	1.00	1.00	1.00
122	.98	.98	.88	.75	.96	.96	.96	1.00	.96	1.00
123	.99	.90	.33^b^	.33^b^	.75	.50	.67	.67	.58	.75
124	.98	.83	.71	.63	1.00	.92	1.00	1.00	.96	.92
Mean for included participants	.98	.95	.64	.73	.96	.87	.92	.83	.91	.96
^a^ fMRI data not acquired due to time constraints. ^b^ Excluded for poor performance. ^c^ Excluded for excessive movement. ^d^ Excluded because English word reading data not acquired or because excluded from analyses of Scan 2.										

## E Brain Regions Differentially Active for English Word and Pseudoword Reading for 20 Participants

**Table:**

Location	Hemisphere	X	Y	Z	No. voxels	*z*-value	Cluster *p*-value
**Pseudoword > Word**							
**Precentral Gyrus**	**Left**	**−40**	**0**	**30**	**2,725**	**5.2**	**<.001**
Rolandic Operculum	Left	−50	8	4			
Precentral Gyrus	Left	−44	−4	40			
Middle Temporal Gyrus	Left	−66	−16	2			
Insula	Left	−28	20	8			
Rolandic Operculum	Left	−58	6	10			
Inferior Frontal Gyrus	Left	−38	32	8			
Middle Temporal Gyrus	Left	−68	−28	8			
IFG p. Opercularis	Left	−56	8	18			
Insula	Left	−38	16	−4			
Insula	Left	−30	30	2			
IFG triangularis	Left	−44	44	12			
Superior Temporal Pole	Left	−52	8	−6			
**Superior Parietal Cortex**	**Left**	**−20**	**−62**	**48**	**1,164**	**5.17**	**.001**
Inferior Parietal Cortex	Left	−38	−42	44			
Superior Parietal Cortex	Left	−26	−54	52			
Middle Occipital Cortex	Left	−28	−70	30			
Inferior Parietal Cortex	Left	−50	−28	48			
**Angular Gyrus**	**Right**	**28**	**−58**	**46**	**447**	**5**	**<.01**
**Inferior Temporal Gyrus**	**Left**	**−44**	**−60**	**−8**	**1,330**	**4.97**	**.001**
Fusiform	Left	−40	−82	−14			
Inferior Occipital Cortex	Left	−34	−90	−8			
Inferior Occipital Cortex	Left	−48	−72	−8			
Inferior Occipital Cortex	Left	−44	−82	−4			
Middle Occipital Cortex	Left	−40	−86	4			
Middle Occipital Cortex	Left	−32	−86	8			
**Inferior Occipital Cortex**	**Right**	**44**	**−84**	**−2**	**507**	**4.66**	**<.05**
Inferior Occipital Cortex	Right	26	−94	−8			
Middle Occipital Cortex	Right	36	−78	12			
Inferior Occipital Cortex	Right	42	−70	−8			
**Supplementary Motor Area**	**Left**	**−4**	**10**	**52**	**544**	**4.28**	**<.01**
Supplementary Motor Area	Right	2	16	46			
**Word > Pseudoword**							
**SupraMarginal Gyrus**	**Left**	**−56**	**−54**	**24**	**2,616**	**5.53**	**<.001**
Middle Occipital Gyrus	Left	−40	−68	24			
White Matter	Left	−38	−54	20			
Angular Gyrus	Left	−44	−54	28			
Angular Gyrus	Left	−44	−60	38			
Middle Temporal Gyrus	Left	−50	−70	16			
White Matter	Left	−36	−48	10			
**Precuneus**	**Left**	**−6**	**−54**	**22**	**8,220**	**5.33**	**<.001**
Precuneus	Left	−8	−62	38			
White Matter	Left	−16	−38	38			
Superior Temporal Gyrus	Right	50	−48	22			
SupraMarginal Gyrus	Right	62	−46	24			
Mid Cingulate	Left	−6	−44	54			
Mid Cingulate	Left	−8	−28	46			
Mid Cingulate	Left	−6	−36	48			
Precuneus	Left	−2	−54	52			
Angular Gyrus	Right	48	−62	24			
Precuneus	Left	−14	−46	50			
Precuneus	Left	−4	−48	8			
Precuneus	Left	−4	−50	38			
Precuneus	Right	6	−56	50			
Precuneus	Right	8	−54	26			
Precuneus	Right	20	−46	20			
Precuneus	Right	14	−44	50			
Precuneus	Left	−16	−54	28			
Mid Cingulate	Left	−4	−16	42			
Middle Temporal Gyrus	Left	−24	−20	34			
**Middle Temporal Gyrus**	**Left**	**−48**	**−6**	**−18**	**716**	**5.24**	**.001**
Middle Temporal Gyrus	Left	−62	−8	−16			
Middle Temporal Gyrus	Left	−50	−18	−16			
**Superior Medial Gyrus**	**Right**	**2**	**60**	**10**	**4,630**	**4.99**	**<.001**
Superior Frontal Gyrus	Left	−16	40	42			
Mid Orbital Gyrus	Left	−4	52	−4			
Mid Orbital Gyrus	Left	−6	32	−14			
Rectal Gyrus	Left	−4	40	−18			
Rectal Gyrus	Right	6	22	−18			
Mid Orbital Gyrus	Left	−10	44	−6			
Middle Frontal Gyrus	Left	−28	24	42			
Anterior Cingulate	Left	−4	50	8			
Middle Frontal Gyrus	Left	−26	30	52			
Superior Medial Gyrus	Left	−8	60	22			
Rectal Gyrus	Left	−2	26	−20			
Superior Medial Gyrus	Right	0	52	26			
Superior Medial Gyrus	Right	6	58	32			
Middle Frontal Gyrus	Left	−20	30	38			
Middle Frontal Gyrus	Left	−28	38	46			
Middle Frontal Gyrus	Left	−38	12	52			
Superior Frontal Gyrus	Left	−12	32	56			
Superior Medial Gyrus	Left	−4	58	32			
Superior Medial Gyrus	Left	−4	48	46			
**Hippocampus**	**Left**	**−20**	**−16**	**−20**	**484**	**4.63**	**.001**
Hippocampus	Left	−26	−24	−14			
Hippocampus	Left	−34	−36	−8			
ParaHippocampal Gyrus	Left	−20	−30	−12			
White Matter	Left	−38	−40	−2			
Hippocampus	Left	−34	−18	−20			
*Note. *Top 20 peaks > 8 mm apart are reported at a threshold of *p* < .001 uncorrected, and *p* < .05 FWE cluster corrected. The regions written in bold text denote the first peak within a cluster.							

## F Brain Regions Active When Learning the Pronunciations (O–P Learning) Versus the Meanings (O–S Learning) of Novel Words Written in Artificial Orthographies, for 18 Participants in Scan 1

**Table:**

Location	Hemisphere	X	Y	Z	No. voxels	*z*-value	Cluster *p*-value
**See-Hear > Hear-Only**							
**Inferior Temporal Gyrus**	**Right**	**46**	**−70**	**−6**	**26,075**	**Inf**	**<.001**
Middle Occipital Cortex	Left	−30	−92	−4			
Middle Occipital Cortex	Left	−38	−90	−4			
Inferior Occipital Cortex	Right	38	−84	−6			
Middle Occipital Cortex	Right	24	−94	6			
Middle Occipital Cortex	Left	−24	−96	0			
Inferior Occipital Cortex	Right	22	−94	−6			
Inferior Occipital Cortex	Left	−22	−90	−8			
Inferior Occipital Cortex	Left	−48	−72	−2			
Middle Occipital Cortex	Left	−42	−86	6			
Inferior Occipital Cortex	Left	−44	−64	−12			
Middle Occipital Cortex	Right	40	−86	14			
**White Matter**	**Right**	**20**	**−30**	**2**	**1,899**	**7.16**	**<.001**
Thalamus	Left	−18	−30	0			
Thalamus	Left	−10	−18	10			
Thalamus	Right	12	−18	10			
White Matter	Left	−12	−18	−8			
**Precentral Gyrus**	**Left**	**−50**	**6**	**28**	**4,891**	**6.90**	**<.001**
Precentral Gyrus	Left	−46	−2	42			
Precentral Gyrus	Left	−36	−8	48			
Superior Frontal Gyrus	Left	−24	−4	54			
IFG p. Triangularis	Left	−52	32	22			
IFG p. Triangularis	Left	−52	36	12			
Supplementary Motor Area	Right	8	8	54			
Supplementary Motor Area	Left	−6	10	56			
Supplementary Motor Area	Left	−4	0	66			
Medial Superior Frontal Gyrus	Left	−8	20	44			
**Precentral Gyrus**	**Left**	**50**	**10**	**32**	**2,708**	**6.42**	**<.001**
Precentral Gyrus	Left	28	−4	50			
Middle Frontal Gyrus	Left	34	−2	62			
IFG p. Triangularis	Left	52	38	14			
Precentral Gyrus	Left	54	0	46			
IFG p. Triangularis	Left	46	22	26			
IFG p. Triangularis	Left	42	28	20			
**OS Learning (see-hear + hear-only) > OP Learning (see-hear + hear-only)**							
**Fusiform Gyrus**	**Left**	**−32**	**−32**	**−16**	**2,381**	**5.12**	**<.001**
Temporal Pole	Left	−30	8	−26			
ParaHippocampal Gyrus	Left	−28	−28	−24			
Temporal Pole	Left	−40	28	−16			
Superior Temporal Gyrus	Left	−42	−4	−14			
Temporal Pole	Left	−48	4	−16			
White Matter	Left	−44	−16	−12			
Fusiform Gyrus	Left	−44	−40	−20			
Temporal Pole	Left	−46	22	−14			
IFG p. Orbitalis	Left	−46	30	−10			
Middle Temporal Gyrus	Left	−52	−20	−16			
White Matter	Left	−32	−2	−18			
**Temporal Pole**	**Right**	**30**	**12**	**−24**	**1,929**	**4.75**	**<.001**
Temporal Pole	Right	34	14	−32			
Insula	Right	42	6	−10			
Amygdala	Right	32	2	−28			
Superior Temporal Gyrus	Right	64	−16	6			
IFG p. Orbitalis	Right	50	30	−6			
IFG p. Opercularis	Right	52	10	12			
Superior Temporal Gyrus	Right	58	0	−4			
IFG p. Orbitalis	Right	42	34	−12			
IFG p. Triangularis	Right	52	40	0			
Middle Temporal Gyrus	Right	52	4	−20			
Superior Temporal Pole	Right	54	16	−4			
**Cerebelum Crus 2**	**Right**	**20**	**−82**	**−36**	**298**	**4.67**	**<.05**
Cerebelum Crus 2	Right	12	−84	−42			
**Superior Frontal Gyrus**	**Left**	**−14**	**34**	**56**	**437**	**4.21**	**<.05**
Middle Frontal Gyrus	Left	−30	28	52			
Superior Medial Gyrus	Left	−2	62	24			
Superior Frontal Gyrus	Left	−24	38	48			
Superior Medial Gyrus	Left	−16	46	46			
Superior Medial Gyrus	Left	−2	40	52			
Superior Medial Gyrus	Left	−6	52	44			
Superior Medial Gyrus	Left	−8	52	32			
Superior Medial Gyrus	Right	4	56	12			
Superior Frontal Gyrus	Left	−12	54	38			
**Inferior Temporal Gyrus**	**Left**	**−60**	**−50**	**−10**	**470**	**4.18**	**<.01**
Middle Temporal Gyrus	Left	−56	−48	−2			
Middle Temporal Gyrus	Left	−48	−46	−2			
**Superior Frontal Gyrus**	**Right**	**28**	**−6**	**370**	**370**	**4.06**	**.01**
Superior Frontal Gyrus	Right	20	−4	70			
Superior Frontal Gyrus	Right	26	4	66			
Superior Frontal Gyrus	Right	26	10	60			
Supplementary Motor Area	Right	14	4	70			
**Middle Temporal Gyrus**	**Left**	**−48**	**−64**	**22**	**249**	**4.02**	**.12**
Middle Occipital Cortex	Left	−44	−78	32			
**White Matter**	**Right**	**10**	**−2**	**−4**	**170**	**3.94**	**<.05**
White Matter	Right	14	−16	−6			
White Matter	Right	8	−26	−4			
**OS Learning (see-hear > hear-only) > OP Learning (see-hear > hear-only)**							
**IFG p. Orbitalis**	**Left**	**−38**	**34**	**−12**	**524**	**5.26**	**<.01**
IFG p. Orbitalis	Left	−40	26	−18			
*Note*. Top 12 peaks > 8 mm apart reported at a threshold of *p* < .001 uncorrected, *p* < .05 FWE cluster corrected. The regions written in bold text denote the first peak within a cluster.							

## G Brain Regions Differentially Active When Retrieving the Pronunciations (O–P Recall) Versus the Meanings (O–S Recall) of Novel Words Written in Artificial Orthographies, for 18 Participants in Scan 1

**Table:**

Location	Hemisphere	X	Y	Z	No. voxels	*z*-value	Cluster *p*-value
**OP Recall (see-think + see-speak) > OS Recall (see-think + see-speak) **							
**IFG p. Triangularis**	**Left**	**−42**	**34**	**12**	**2,701**	**6.13**	**<.001**
IFG p. Opercularis	Left	−56	10	28			
Precentral Gyrus	Left	−48	0	44			
IFG p. Triangularis	Left	−52	22	−2			
**Cerebelum**	**Right**	**20**	**−68**	**−50**	**718**	**5.11**	**.001**
Cerebelum	Right	26	−66	−24			
**Posterior-Medial Frontal**	**Left**	**−4**	**8**	**60**	**221**	**4.44**	**.06**
Posterior-Medial Frontal	Left	−10	10	68			
**Postcentral Gyrus**	**Left**	**−56**	**−20**	**32**	**814**	**4.33**	**<.01**
Inferior Parietal Lobule	Left	−42	−36	36			
White Matter	Left	−32	38	38			
Inferior Parietal Lobule	Left	−56	−28	42			
Inferior Parietal Lobule	Left	−28	−46	46			
Inferior Parietal Lobule	Left	−46	−40	56			
Inferior Parietal Lobule	Left	−42	−38	46			
Inferior Parietal Lobule	Left	−52	−34	52			
Inferior Parietal Lobule	Left	−34	−46	56			
*Note*. Top 12 Peaks > 8 mm apart are reported at a threshold of *p* < .001 uncorrected, and *p* < .05 FWE cluster corrected. The regions written in bold text denote the first peak within a cluster.							

## H Results of Post-Hoc Tests From an ANOVA conducted on Correct See-Think Trials in the Reading Aloud Task, With the Factors Lexicality (Trained vs. Untrained) and Training Focus (OP vs. OS). Data are From 20 Participants in Scan 2 at the End of Training

**Table:**

Location	Hemisphere	X	Y	Z	No. voxels	*z*-value	Cluster *p*-value
**Trained > Untrained**							
**SupraMarginal Gyrus**	**Left**	**−52**	**−48**	**36**	**4,191**	**5.69**	**<.001**
Superior Temporal Gyrus	Left	−50	−28	20			
Middle Temporal Gyrus	Left	−62	−44	−2			
Angular Gyrus	Left	−44	−56	34			
Middle Temporal Gyrus	Left	−62	−26	−16			
Angular Gyrus	Left	−38	−52	28			
Angular Gyrus	Left	−36	−62	28			
Middle Temporal Gyrus	Left	−56	−18	−18			
Middle Temporal Gyrus	Left	−52	−30	−8			
Angular Gyrus	Left	−46	−66	30			
Insula Lobe	Left	−38	−14	14			
Middle Temporal Gyrus	Left	−58	−20	−6			
**Middle Frontal Gyrus**	**Left**	**−32**	**60**	**14**	**614**	**5.46**	**<.05**
Middle Orbital Gyrus	Left	−38	54	−10			
Middle Frontal Gyrus	Left	−38	58	0			
**Rolandic Operculum**	**Right**	**52**	**−20**	**22**	**3,824**	**5.18**	**<.001**
SupraMarginal Gyrus	Right	50	−26	28			
SupraMarginal Gyrus	Right	60	−26	24			
Inferior Parietal Lobule	Right	48	−48	44			
Angular Gyrus	Right	38	−52	32			
Superior Temporal Gyrus	Right	54	−36	20			
Inferior Parietal Lobule	Right	44	−56	44			
Middle Temporal Gyrus	Right	64	−36	2			
Middle Temporal Gyrus	Right	60	−44	2			
Angular Gyrus	Right	52	−52	36			
Middle Temporal Gyrus	Right	62	−30	−12			
Insula Lobe	Right	34	−18	6			
White Matter	Right	36	−16	34			
Superior Temporal Gyrus	Right	48	−46	12			
Rolandic Operculum	Right	56	0	8			
Superior Temporal Gyrus	Right	70	−24	0			
**Middle Frontal Gyrus**	**Left**	**−32**	**16**	**50**	**793**	**5.03**	**<.01**
Middle Frontal Gyrus	Left	−40	26	44			
**Middle Frontal Gyrus**	**Right**	**38**	**14**	**48**	**506**	**4.87**	**<.01**
Middle Frontal Gyrus	Right	38	28	40			
**Cuneus**	**Right**	**6**	**−90**	**16**	**2,361**	**4.85**	**<.001**
Cuneus	Right	10	−84	28			
Cuneus	Right	12	−80	36			
Cuneus	Left	−10	−78	34			
Cuneus	Left	−8	−86	26			
Lingual Gyrus	Left	−6	−76	−4			
Lingual Gyrus	Right	10	−72	−2			
Lingual Gyrus	Right	6	−80	0			
Lingual Gyrus	Right	10	−62	−6			
Precuneus	Right	6	−56	36			
Lingual Gyrus	Right	22	−76	2			
Lingual Gyrus	Right	14	−52	−2			
**Precuneus**	**Left**	**−10**	**−46**	**54**	**285**	**4.39**	**<.05**
Precuneus	Left	−12	−44	42			
Precuneus	Left	−14	−54	36			
White matter	Left	−14	−12	38			
**White matter**	**Left**	**−30**	**−18**	**28**	**186**	**3.69**	**<.05**
Midcingulate	Left	−12	−4	44			
Midcingulate	Left	−8	−20	36			
White matter	Left	−20	−4	32			
White matter	Left	−28	−6	28			
**Untrained > Trained**							
**Precentral Gyrus**	**Left**	**−48**	**0**	**38**	**1,358**	**6.52**	**<.001**
Precentral Gyrus	Left	−48	4	26			
**Posterior-Medial Frontal**	**Left**	**−6**	**4**	**60**	**429**	**6.14**	**<.05**
Posterior-Medial Frontal	Left	−8	16	46			
Posterior-Medial Frontal	Right	8	8	54			
**Inferior Occipital Gyrus**	**Left**	**−40**	**−66**	**−10**	**2,040**	**6.06**	**<.001**
Inferior Temporal Gyrus	Left	−46	−58	−10			
Inferior Occipital Gyrus	Left	−34	−86	−6			
Middle Occipital Gyrus	Left	−32	−88	14			
Superior Parietal Lobule	Left	−22	−58	52			
Middle Occipital Gyrus	Left	−28	−80	22			
Middle Occipital Gyrus	Left	−24	−68	36			
Middle Occipital Gyrus	Left	−26	−74	30			
**Inferior Occipital Gyrus**	**Right**	**42**	**−70**	**−10**	**1,030**	**5.53**	**<.001**
Middle Occipital Gyrus	Right	38	−84	10			
Inferior Occipital Gyrus	Right	32	−86	−6			
**IFG p. Triangularis**	**Left**	**−42**	**32**	**18**	**1,018**	**5.24**	**.001**
IFG p. Triangularis	Left	−38	26	4			
**Inferior Parietal Lobule**	**Left**	**−42**	**−38**	**44**	**369**	**4.56**	**<.01**
SupraMarginal Gyrus	Left	−62	−22	32			
Inferior Parietal Lobule	Left	−54	−30	40			
**OS > OP focus**							
**Lingual Gyrus**	**Right**	**24**	**−90**	**−6**	**2,406**	**5.53**	**<.001**
Calcarine Gyrus	Right	20	−94	2			
Inferior Occipital Gyrus	Right	38	−80	−10			
Middle Occipital Gyrus	Right	40	−86	16			
Inferior Occipital Gyrus	Right	42	−72	−10			
Inferior Occipital Gyrus	Right	32	−86	−6			
Middle Occipital Gyrus	Right	28	−94	12			
Middle Occipital Gyrus	Right	38	−88	4			
Inferior Temporal Gyrus	Right	52	−58	−10			
Inferior Temporal Gyrus	Right	54	−50	−18			
Middle Temporal Gyrus	Right	50	−74	10			
Middle Occipital Gyrus	Right	30	−84	32			
**Inferior Occipital Gyrus**	**Left**	**−32**	**−88**	**−8**	**2,391**	**4.99**	**<.001**
Inferior Occipital Gyrus	Left	−22	−92	−8			
Inferior Parietal Lobule	Left	−28	−74	42			
Middle Occipital Gyrus	Left	−30	−92	18			
Inferior Temporal Gyrus	Left	−50	−60	−8			
Middle Occipital Gyrus	Left	−18	−98	6			
Middle Occipital Gyrus	Left	−30	−78	24			
Middle Occipital Gyrus	Left	−32	−86	24			
Superior Parietal Lobule	Left	−18	−66	48			
Inferior Temporal Gyrus	Left	−44	−66	−10			
Middle Occipital Gyrus	Left	−22	−98	14			
Middle Occipital Gyrus	Left	−26	−96	2			
**Middle Frontal Gyrus**	**Left**	**−22**	**4**	**48**	**2,192**	**4.4**	**<.001**
Posterior-Medial Frontal	Left	−6	16	48			
Superior Frontal Gyrus	Left	−22	2	58			
Middle Frontal Gyrus	Left	−42	8	36			
Middle Frontal Gyrus	Left	−32	2	56			
Posterior-Medial Frontal	Right	8	12	50			
Superior Frontal Gyrus	Left	−22	12	56			
Posterior-Medial Frontal	Right	4	−2	48			
Middle Frontal Gyrus	Left	−48	18	36			
Superior Frontal Gyrus	Left	−14	2	52			
IFG p. Triangularis	Left	−48	28	20			
*Note.* Main effects of lexicality (untrained vs. trained) and training focus (O–S vs. O–P) were obtained. Top 12 peaks > 8 mm apart are reported at a threshold of *p* < .001 uncorrected, *p* < .05 FWE cluster corrected. The regions written in bold text denote the first peak within a cluster.							
